# Earliest evidence of caries lesion in hominids reveal sugar-rich diet for a Middle Miocene dryopithecine from Europe

**DOI:** 10.1371/journal.pone.0203307

**Published:** 2018-08-30

**Authors:** Jochen Fuss, Gregor Uhlig, Madelaine Böhme

**Affiliations:** 1 Department of Geoscience, Eberhard-Karls-University Tübingen, Tübingen, Germany; 2 Senckenberg Centre for Human Evolution and Palaeoenvironment (HEP), Tübingen, Germany; 3 Department of Chemistry and Food Chemistry, Technical University Dresden, Dresden, Germany; Oregon State University, UNITED STATES

## Abstract

The formation of dental caries is mainly caused by dietary habits and therefore, may contain information for dietary reconstructions of fossil hominids. This study investigates the caries lesion in the 12.5 Ma old type specimen of *Dryopithecus carinthiacus* Mottl 1957 (Primates, Hominidae) from St. Stefan (Austria). Potential food sources are identified on associated palynological data, which allow conclusions about food quality, sugar availability and the hominid metabolism during the Middle Miocene. Using micro computed tomography (μCT) and scanning electron microscopy (SEM) we provide a detailed analysis and characterization of the individuals’ caries type. Its lesion is compared with a dataset of 311 wild chimpanzees, indicating morphological and etiological differences in caries formation between both species. The affected molar of *D*. *carinthiacus* reveals features known from severe dental caries in humans: (1) Cavitation with steep walls and smooth surface; (2) Reparative dentine at the roof of the pulp chamber; (3) Sclerotic dentine below the cavitation; (4) Association with dental calculus and (5) Unilateral usage of the healthy right tooth row. Its advanced primary caries, initiating on the intact enamel surface, indicates a frequent intake of highly cariogenic sugar-rich fruits, which likely exceeds the frugivory of extant chimpanzees. This finding corresponds with the associated palynological record, which infers a habitat with nearly year-round supply (9–10 months/year) of high quality foods (>carbohydrates; < fibers). Our conclusions challenge the model of a step-wise increase in dietary quality during hominid evolution and support the uricase hypothesis, which discusses the hominid autapomorphy of a fructose-based fat accumulation for periods of starvation. This model receives further validation by the identification of soft-tissue preservation, interpreted as fossilized white adipose cells, in the articulated hominid skeleton of *Oreopithecus bamboli* from Italy.

## 1. Introduction

**Fig 1:**
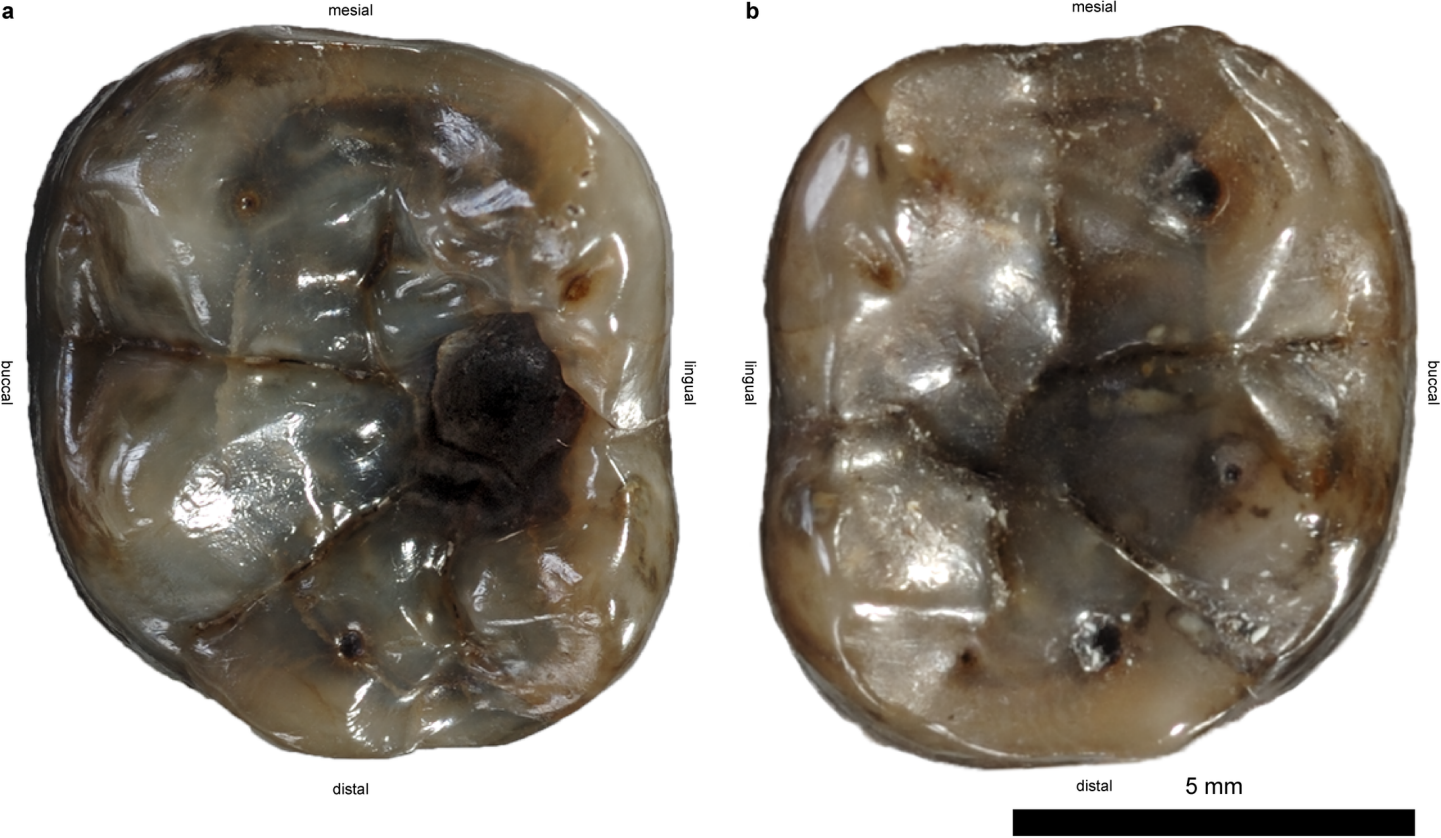
Lower first molars of the type specimen of *D*. *carinthiacus* (LMK-Pal 5508) in occlusal view. **a**, Carious left m1. **b**, right m1 without caries lesion, graphically isolated from mandibular bone.

Dental caries is a prevalent disease in current societies, reflecting the oral health and dietary conditions of an individual. Although scarce in the fossil record, its occurrence can also give rare insights into the dietary preferences of extinct taxa [1]. Rare records of fossil hominid dental caries are restricted to hominins from the Pleistocene epoch [2–4]. Here we describe a considerably older caries lesion from the Middle Miocene stem-hominine *Dryopithecus carinthiacus* (Fig 1) from St. Stefan (Lavanttal Basin, Carinthia, Austria). It is the earliest documented caries disease in hominids dating back to the Early Sarmatian (Serravallian, late Middle Miocene) at 12.5 Ma. Its primary formation on the intact enamel surface indicates a highly cariogenic diet of *D*. *carinthiacus*, supposedly rich in sugar-rich fruits. For comparison, we developed a database of caries type frequencies for a large population of wild chimpanzees (*Pan troglodytes verus*, n = 311), known as the most frugivorous extant great apes. Based on palynological data from St. Stefan, we draw inferences about the seasonal availability of fruits and honey, and discuss the potential diet of *D*. *carinthiacus* in the framework of hominid metabolism. Our results provide conclusions about the dryopithecine metabolism, supporting the uricase hypothesis [5, 6] and offering new insights into the dietary evolution of early hominids.

### 1.1. Hominoid dietary evolution

Hominoids occupied the European continent for about 10 million years during which continental environments, vegetation, and climate changed dramatically. They initially entered the continent at 15.9 Ma (earliest Langhian; recalibrated according to [7]), when it was a paratropical rainforest environment at the start of the Miocene Climatic Optimum [8]. Their last appearance is recorded at 7.175 Ma (earliest Messinian) from a savannah biome of post-Pikermian time [9, 10] as well as from the late Messinian (5.7 Ma) Trachilos footprints [11], suggesting substantial shifts in hominid diet and physiology during their presence in Europe.

Extant hominids show a wealth of dietary adaptions that range from diverse vegetal diets (leaves, fruits, tubers) to animal prey (invertebrates and small vertebrates). All living primates are largely territorial and thus, need to cope with the local food resources [12]. Some taxa are specialists that concentrate on certain resources. Today, these dietary specialists mainly occur in low latitudinal forest habitats that show rather low seasonal changes (mainly in rainfall) and a high plant and animal diversity [13]. The high diversity implies high competition for resources, but also allows a specialization on certain food items to reduce dietary competition. In habitats that are more temperate the diversity and interspecies competition is less [14]. However, the availability of resources is subject to larger fluctuations due to seasonal changes in temperature and day length. Primates adapted to such conditions have a larger home range [14] and are able to use a wider, seasonally varying food spectrum.

Likewise, the fossil hominoid record shows similar and even more extreme adaptions to a wider range of resources [15]. Studies in dentognathic morphology of fossil hominoids suggest a general shift from soft to hard object feeders during the Early to Late Miocene [16]. This shift in anatomy reflects the underlying major changes in habitat and climate with which hominoids had to cope with [8, 10]. The early phase of primate evolution, from Eocene to Oligocene, was represented by small insectivores, followed by folivores with sharp-ridged teeth that were adapted to chop leaves and probably small invertebrates [16]. During the early Miocene, the broader cusps of early hominoids like *Proconsul* were well suited to pulp fruits and might have been more suitable for a slightly larger food spectrum. However, the lack of shearing blades reduced their ability to adequately process leaf tissue [17]. Similar to their folivorous precursors, their thin enamel, the small teeth and the gracile mandibles were not suitable for abrasive food and hard mastication. However, during the late Early Miocene, afropithecine taxa (*Afropithecus*, *Heliopithecus*) developed larger jaws [18] and for the first time thick enameled teeth [19]. During the Middle Miocene, thick enameled equatorines (*Equatorius*, *Nacholapithecus*) and kenyapithecines (*Griphopithecus*, *Kenyapithecus*) populated East Africa, Anatolia and Europe [8, 20, 21]. Tooth morphology, enamel thickness and microstructure indicate a diet composed of hard and abrasive food [16, 22, 23]. During the Middle to Late Miocene of Asia, sivapithecines enhanced this adaption with the thick enameled and low-cusped teeth, probably an adaption to intensified seasonality and more opened habitats [16]. Contemporaneously, some of the diverse European dryopithecines (*Dryopithecus*, *Hispanopithecus*, *Rudapithecus*) from early Late Miocene showed rather thin enameled teeth with broad cusps, reminding of the dentition of the largely frugivorous extant chimpanzee [15, 24]. Microwear analyses of incisors and molars infer an intermediate position between frugivores/mixed feeders and hard-object feeders [22, 25]. In contrast, the Middle Miocene dryopithecines *Pierolapithecus* and *Anoiapithecus* are characterized as arboreal hard-object feeders, based on the molar microwear and their thicker enamel [22, 26].

The shift towards a hard-object consumption clearly increased during the later parts of the Late Miocene, particularly in Eurasian hominids like *Ankarapithecus*, *Ouranopithecus* and *Graecopithecus* [9, 15, 27, 28]. Their thick-enameled and partially megadont molars enabled them to masticate on fibrous underground foods and highly abrasive grit that becomes ubiquitous with the near-ground eating and the increasing aridity during the Tortonian and Early Messinian [10, 22, 25].

### 1.2. The Hominid fructose and uric acid metabolic system

Dietary changes during the hominoid evolution are still visible in the physiology of extant hominids, including our own species. An important impact on our current metabolic system might have been the Middle Miocene introduction of a fructose-based diet:

The metabolic pathways of fructose and uric acid are intimately connected. Fructose naturally occurs as monosaccharide in food like fruits and honey, or together with glucose as the disaccharide saccharose (sucrose). Once ingested and resorbed, fructose is instantly converted into fructose-1-phosphate through depletion of ATP to ADP/AMP, resulting in a lack of ATP. ADP/AMP is then, over few more steps, converted into uric acid [29]. This process is accompanied by systemic oxidative stress [30, 31]. Uric acid itself is a high effective anti-oxidant [32] and stimulates the further degradation of fructose. According, there exists a positive feedback interdependency between fructose and uric acid. Both block the degradation of fatty acids (fructose through the lack of ATP, uric acid inhibits an essential key enzyme) and both rather stimulate lipogenesis [33]. In fact, a fructose-rich diet leads to significant more body fat gain in comparison to an iso-caloric fructose-free diet [34, 35]. High uric acid serum levels also lead to an elevated blood pressure. Furthermore, it is discussed that individuals with elevated uric acid levels may be more active, excitement seeking and venturous in comparison to individuals having lower uric acid levels [36]. In many organisms, uric acid is depleted by the enzyme uricase. In early hominids, however, several pseudogenization events of uricase appeared, finally resulting in a knockout of uricase function and thus permanent elevated serum uric acid levels around 15 Ma [37]. It is hypothesized that the uricase knockout in hominids may have provided a significant survival advantage for upcoming times of food shortage [5]. In an environment where fructose and other short chain carbohydrates are only seasonally available, high uric acid levels allow an accelerated accumulation of fat stores and a more effective utilization of the attainable nutrition. Elevated blood pressure as well as increased activity and venturesomeness may provide an additional advantage for early hominids. Furthermore, it is discussed that high uric acid serum levels may be associated with a higher intelligence [38, 39]. Anyway, it is widely accepted that uric acid is a highly potent neuroprotective agent [38, 40, 41]. Due to its ability to penetrate the blood-brain-barrier, uric acid is considered to decrease the risk of neurodegenerative diseases [38, 42–44]. The fact that a fructose-rich diet rapidly increases fat stores is also found in the seasonal changing food pattern of hibernating and migrating animals [45]. For example, in autumn, migrating songbirds switch from insect or seed based food to fruits to accelerate the accumulation of body fat, which is supposed to be the main energy reserve during long distance migration [46].

### 1.3. Caries formation

Due to its common occurrence in modern humans, the process of caries formation is intensively studied in both, extant and extinct humans (e.g. [47, 48]). Caries is the demineralization of dental hard tissue (enamel, cementum and dentine) due to organic acids formed by bacteria in dental plaque [49]. Usually, these pathogenic bacteria (e.g. *Streptococcus mutans*) are already present in the healthy oral microflora. The dental hard tissue is only affected, if the homeostasis of the oral flora is disturbed. A diet rich in carbohydrates and a low oral pH may cause this disequilibrium. The anaerobic metabolism of particular bacteria, such as *S*. *mutans*, converts sugar into organic acids. These acids are lowering the pH of the plaque biofilm and the saliva and demineralize the calcium hydroxyapatite in particular sites of the dentition. Dental plaque preferentially adheres in the interdental spaces, gingival margins and occlusal grooves causing caries lesions typically at these sites. Without treatment, the dissolution of dental hard tissue progresses and leads to the formation of deep cavities [48]. A deep cavity exposing the dentine is the last stage of a carious enamel lesion. In human teeth it needs about 3–4 years to reach this stadium [50], albeit this penetration time can be far more variable depending on the individual risk status [51].

In modern humans, caries mostly appears in children and mature adults; young adults are less affected. In children occlusal and interstitial caries dominates, while adults are mainly affected by cervical caries [48, 52]. In adult humans, as well as in non-human primates, the prevalence of caries increases with age [53]. Partially, this is related to the nature of caries as a progressive disease. The proceeding cavitation can pause, but it cannot be restored. Thereby, cavitation increases with age if not treated. Another cause for age-related caries lesions is the way caries is initiated. The formation of caries can be classified in two types [53]: The primary caries initiates on the intact enamel surface. It is common in captured animals and humans, but rare in the wild. The secondary caries is prevalent in wild animals and relates to enamel fractures and/or tooth wear, which proceeds with age. Thereby, cariogenic bacteria may infiltrate the exposed dentine and damage the enamel on a larger area. In turn, a high wear rate might remove occlusal caries, if it develops slowly [54].

### 1.4. Cariogenic diet

A cariogenic diet is characterized by a high content of quickly fermentable carbohydrates (e.g. fructose, glucose, sucrose, or cooked starch) and the easy adherence on tooth surfaces. The frequent consumption of such substances lowers the pH of the saliva and thereby, favors the multiplication of cariogenic oral bacteria at the expense of beneficial strains [50].

A key cause of today’s caries lesions in humans is our diet that is rich in free sugars. Beside these refined sugars, the naturally present sugars (e.g. in fruits or honey) and starch-containing foods are supposed to be cariogenic. However, the role of starch as a cariogenic or co-cariogenic substance is complex. The starch granules first need a mechanical and heat treatment to become effectively cariogenic [50, 55]. This process of gelatinization releases the glucose polymers amylose and amylopectin, which makes them susceptible to the enzymatic breakdown [55]. As an elaborated food preparation is required for this, starch can largely be ignored as a major source of caries in wild animals.

### 1.5. Caries in fossil hominins

Caries is an ancient disease, not only restricted to the human clade, but also known from a variety of extant and extinct animals. Early records of potential carious destruction of dental hard tissue come from diverse groups of Palaeozoic and Mesozoic animals [56, 57] and Neogene mammals like bovids and bears [1, 58]. However, the incidence in wild animals is very rare compared to modern humans [53, 59, 60]. Dental caries is one of the most prevalent diseases in present societies. Its widespread emergence is a young phenomenon, initiated by the industrial era and mainly associated with the increased consumption of sugar-rich food [61]. Caries is a common oral disease of humans since about 10,000 years, associated with the shift from hunter-gatherers to agriculturalists [4, 47, 62, 63]. Until the Late Paleolithic, early modern humans rarely had caries [64–66]. Older caries lesions are documented in ~60ka old *H*. *neanderthalensis* (e.g. [67, 68]) albeit they are not as prevalent as in modern humans [62]. The previously earliest caries lesions in hominids are described in *Homo rhodesiensis* (ca. 650ka-160ka) [62, 69], *Gigantopithecus blacki* (1.2Ma-310ka) [70, 71], *Homo erectus*, *Paranthropus robustus* (ca. 1.5 Ma) [72], and early *Homo* from Dmanisi (1.77 Ma) [66], all from the Pleistocene epoch.

### 1.6. Caries in non-human primate dentition

Most studies on caries are aimed for the humans’ dental health, but even if caries is mostly reported from hominin teeth, it also affects non-human dentition [53, 60, 73]. Numerous studies examined the formation of caries on a variety of laboratory animals (e.g. rodents, lagomorphs, primates [53]). However, these experiments are designed to simulate the human conditions and usually, cannot be transferred to wild animals. Further, there are indications that captured/domestic animals and animals living close to human settings may tend to show a higher incidence of caries compared to the wild ones [53, 60]. This suggests human diet and waste disposal as main culprits for their caries formation. Another possibility is the transmission of zoonotic oral bacteria from humans to animals [59]. In the wild, animals show considerably less caries lesions than humans. Beside dietary differences, other factors like a more alkaline saliva, a differing oral microflora and/or tooth surfaces less susceptible to plaque formation may be related to the rare occurrence in wild animals [53]. Experiments on various laboratory animals show that their teeth may generally be prone to primary caries, but heavy dental wear, a short life span and/or a frequent tooth change may additionally prevent its formation [53]. Supposedly, these inhibiting processes are intensified under natural conditions.

The occurrence and frequency of caries in extant primates is reported in several studies, which are largely covered and summarized in Miles and Grigson [53]. Among extant great apes, caries lesions mainly occur in chimpanzees, whose diet is rich in fruits. The folivorous gorillas are less affected [48, 53]; *Pongo*, which is intermediate in diet between *Pan* and *Gorilla* shows an intermediate caries frequency [74]. Crovella and Ardito [60] showed that in adult wild primates (Prosimii, Platyrrhini, and Catarrhini), almost every second individual has dental calculus, while only 7.4% have caries. Extant great apes can have occlusal caries, but in contrast to the primary occlusal caries prevailing in modern human teeth, wild great apes generally show severe secondary occlusal caries that is initiated by enhanced dental wear or fractures. The occurrence of severe interstitial/cervical caries is even more frequent, albeit still rare [53, 74]. While primary caries is so far unreported in fossil great apes, the previously earliest evidence of dental calculus in hominids comes from the Miocene *Sivapithecus sivalensis* from the Siwaliks of NW Pakistan [75, 76] and dates back to ~8.7–9.3 Ma.

## 2. St. Stefan

### 2.1. Geological setting, stratigraphy and age

The hominid fossil locality St. Stefan is situated in the Lavanttal Basin (Carinthia, Austria), at the western margin of the Central Paratethys realm. Due to the underground coal mining till 1968, several vertebrate fossils were exposed including the type specimen of *D*. *carinthiacus* [77]. The Lavanttal Basin covers an area of about 1000 km^2^. The geological structure, sedimentology and stratigraphy of this area are well studied (e.g. [78–81]). The alpine mountain ranges Koralpe and Saualpe confine the southern part of the Lavanttal Basin (including the St. Stefan sub-basin); the Packalpe and the Seetaler Alps confine the northern part. These bordering mountains are the visible outcrops of an underlying synclinal structure that forms the crystalline bedrock of the Lavanttal Basin. Tectonics and sediments divide the southern Lavanttal in two areas [79]: The ‘Mulden’ area (St. Stefan sub-basin (N) and Andersdorf-Ettendorf sub-basin (S)) and the ‘Granitztal’-beds.

The deposits of the St. Stefan sub-basin comprise marine, brackish and freshwater sediments (marls, sands and gravels) of Middle to Late Miocene age (?Karpatian, Badenian to Pannonian), intercalated by productive coal seams. As part of the Central Paratethys, the Lavanttal Basin underwent considerable tectonic, environmental and climatic changes [82, 83]. During the Late Badenian, the Central Paratethys got largely isolated from the Tethys Ocean, but a narrow connectivity remained at the Slovenian strait [84, 85]. The base of the Sarmatian, dated to 12.65 Ma [86] was characterized by a dramatic extinction event of marine taxa [87] initiated by the tectonically and eustatic driven full connection to brackish waters of the Eastern Paratethys [86]. Restricted oceanic connectivity and fluctuations in humidity during the Sarmatian [83] resulted in a changing Paratethyan water chemistry up to hypersaline and alkaline conditions [88]. Short-term marine transgressions and Eastern Paratethyan connectivity introduced mollusc and foraminifera assemblages that allow a biostratigraphic zonation of the Sarmatian in the Central Paratethys [89, 90].

**Fig 2:**
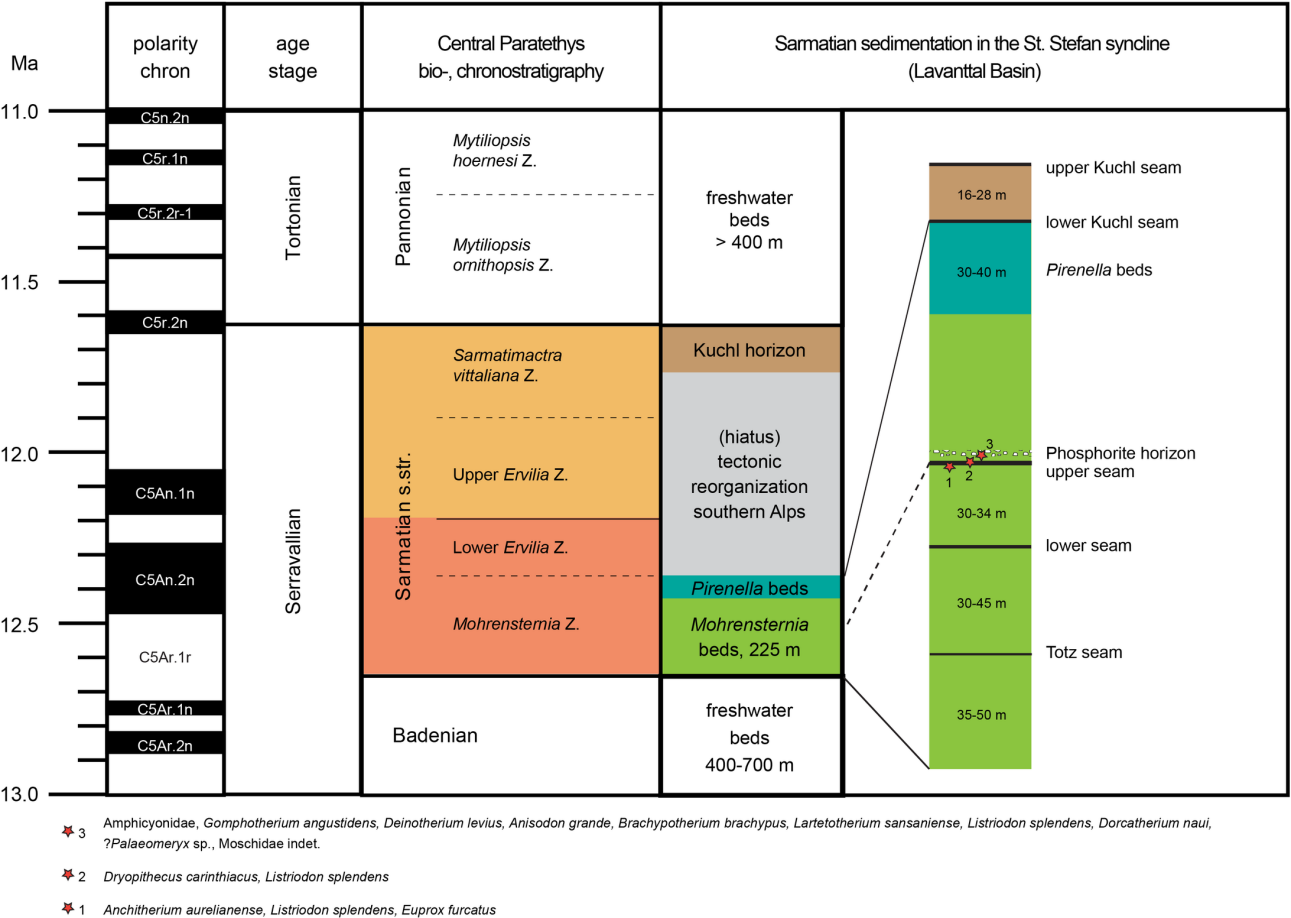
Sarmatian sedimentation and stratigraphy in the St. Stefan syncline (Lavanttal Basin, Carinthia) after Beck-Mannagetta [79] and Papp [89]. Fossil mammal assemblages around the ‘upper seam’ according to [77, 91–94].

The Sarmatian sediments in the Lavanttal Basin intercalate between thick Late Badenian and Early Pannonian freshwater deposits (Fig 2). Three lithologic units and five brown-coal seams characterize the nearly 300 m thick Sarmatian sequence. Both lower units, the *Mohrensternia* beds and the *Pirinella* beds, compose of lacustrine sediments containing brackish intervals with foraminifers and mollusks correlating to the earliest Sarmatian *Mohrensternia* bio-zone [89]. Three coal seams (Totz seam, lower, and upper seam) developed during marginal lacustrine conditions in the *Mohrensternia* beds. The *Pirenella* beds were followed by an erosional phase without depositions [79]. This phase of tectonic reorganization in the southern Alps ended with the discordant deposition of the two Kuchl seams and accompanied sediments, which are assigned to the Late Sarmatian [79].

During underground coal mining activities, the sedimentary sequence of St. Stefan has provided three vertebrate bearing horizons within an only eight meter thick sequence (Fig 2 and [77, 91–93]): (1) *lower bed*, (2) *upper seam*, and (3) *phosphorite* (nodule) *horizon*.

The lower bed is positioned directly below the upper seam and consists of marl. Its uppermost part revealed findings of three mammalian species: *Anchiterium aurelianense* (Equidae), *Listriodon splendens* (Suidae) and *Euprox furcatus* (Cervidae). The hominid *Dryopithecus carinthiacus* and *Listriodon splendens* were found within the upper coal seam. Further vertebrate fossils (Amphicyonidae, *Gomphotherium angustidens*, *Deinotherium levius*, *Anisodon grande*, *Brachypotherium brachyphus*, *Lartetotherium sansaniense*, *Listriodon splendens*, *Dorcatherium naui*,?*Palaeomeryx* sp., Moschidae indet. and *Testudo* sp.) came from the phosphorite horizon about 4 meters above the upper seam [77, 91–93].

With a thickness of 260m, both lower units (*Mohrensternia* and *Pirenella* beds) cover large parts of the Early Sarmatian between 12.65–~12.35 Ma (*Mohrensternia* Zone). This gives a sedimentation rate of 0.87m/ky. Assuming a continuous sedimentation [79], the age of the three vertebrate horizons, which are ~110m above the base of Sarmatian, can be approximated to ~12.5 Ma. Thereby, St. Stefan provides with *D*. *carinthiacus* the stratigraphic earliest representative of the dryopithecine clade.

### 2.2. Palaeo-vegetation of St. Stefan

The palaeobotanic records from the Sarmatian of the Lavanttal comprise leaves [95], spores and pollen studied from the phosphorite nodule horizon [78]. We refer this palynologic data to the *Dryopithecus* environment, because the phosphorite horizon is situated about four meters above the upper seam, which suggests a younger age, compared to the fossil ape, by only a few thousand years. The palynoflora is very diverse and comprises more than 170 taxa [78, 96–99]. The high taxonomic resolution is due to exceptional 3D pollen-preservation in phosphorite nodules, enabling the identification of taxa using both light microscopy and scanning electron microscopy [96]. These botanical data point to mixed mesophytic forests in the coastal lowlands [78], dominated by Fagaceae (evergreen and deciduous *Quercus*, *Fagus*, *Castanea*) and Juglandaceae (*Carya*, *Pterocarya*, *Engelhardia*, *Juglans*).

## 3. Material and methods

### 3.1. *Dryopithecus carinthiacus*, Mottl 1957

The studied object LMK-Pal 5508 is the type specimen of the Middle Miocene hominid *Dryopithecus (fontani) carinthiacus* Mottl 1957 from St. Stefan (Carinthia, Austria). It is a partial right mandible with p3, p4 and m1 and isolated right i1, left c, p3, p4 and m1 of the same individual. A partial right m2, reported in Mottl [77], is missing today. The focus of this study is on the carious left m1. The holotype is housed in the collection of the ‘Landesmuseum für Kärnten’ in Klagenfurt am Wörthersee (Austria).

#### SEM-imaging

A detailed analysis of the caries morphology was performed to exclude other destructions of dental hart tissue that might have a similar appearance like attritional or post-mortem defects. Further, potential calculus deposits on the tooth surface are identified based on their cavernous and spherical surface structure. Therefore, the tooth surface of the studied object was examined with stereo light microscopy (LM) and scanning electron microscopy (SEM). Before SEM imaging, the specimen was mechanically cleaned with a soft brush and water in order to remove loose particles from surface. The cleaned specimen was placed on an aluminum stub and imaged in the LEO-1450VP scanning electron microscope at the Department of Geoscience (University of Tübingen, Germany). The specimen was left uncoated due to curatorial reasons.

#### Laserscanning microscopy

Further surface features including microwear were examined with the laser-scanning microscope Keyence VK-X 130K at the Department of Geoscience (CCA-BW, University of Tübingen, Germany). The device has a confocal aperture and a 658nm red laser. The wear facets 9 and 10 of the right and left m1 were scanned using a 20x objective that covered an area of 698 x 524 μm, 1024 x 768 pixel and a vertical resolution of 0.005 μm. The numerical system of wear facets follows [100, 101]. Image processing was done with the Keyence software VK-Analyzer 3.8. The photosimulations were reduced to an area of 400 x 400 μm and were constricted to the laser intensity. Thereby, the actual surface topology was visualized in grey scale without being overlain by colors, textures and light incidence.

#### μCT scanning

Internal dental structures of the left m1 of LMK-Pal 5508 were visualized based on X-ray microtomographic (μCT) scans. The isolated tooth was scanned with the GE Phoenix v|tome|x s μCT scanner at the Institute for Archaeological science at the University of Tübingen. The μCT-scan was performed at 170 kV and 170 μA with a resolution of 32.56 μm. No corrections for artefacts were necessary. The conversion into 3D volumes and the segmentation work has been performed in Avizo 8.0. The different materials (enamel, dentine, and pulp chamber) were semi-automatically segmented applying surface determination, region growing and masking tools. The linear measurements were done on the virtual 3D model with the 3D measuring tool in Avizo 8.0.

### 3.2. *Pan troglodytes verus*, Schwarz 1934

The comparative samples of *Pan troglodytes verus* came from the Senckenberg skull collection of Liberian chimpanzees in Frankfurt a. M. (Germany). The observed specimens were wild caught individuals including juveniles and adults. Only specimens with at least one erupted and unfractured tooth were used for the counting. Thereby, from a total of n = 365 observed individuals (S1 Table), there were n = 311 individuals (32 juveniles and 279 adults) with complete or partial dentition. As many individuals featured pre- and post-mortem loss of teeth, we did not count the caries frequency per individual, but the frequency per total number of observed teeth. In total n = 3259 erupted teeth were sighted (n = 2890 permanent; n = 369 deciduous).

We differentiated between three types of caries cavities: (1) interstitial/cervical, (2) secondary occlusal and (3) primary occlusal. Caries cavities in the dental root or enamel, which are positioned in the interdental spaces or on the cervices were classified as interstitial/cervical. Secondary occlusal cavities were defined as being positioned on the occlusal enamel/dentine surface and being related to heavy occlusal wear or fractures. Primary occlusal caries initiates on intact, largely unworn occlusal enamel surfaces.

The presence of caries was determined macroscopically and with a hand lens. Only advanced caries lesions that already formed cavities in the dental hard tissue were counted, as earlier stages are difficult to identify by this technique. Further, there could be a slight underestimation of interstitial caries, as tight interdental spaces might have hidden the lesions from the superficial inspection. As the focus of this article is on the primary caries of *D*. *carinthiacus*, we did not apply more extended analyses on hidden interstitial caries here.

### 3.3. Sugar content of food sources

To evaluate the sugar contents (fructose, glucose, saccharose) of fruit-producing plant taxa identified in the palynological record of St. Stefan, we consulted nutritional databases for common fruit types [102], whereas those contents for rarely consumed fruits were extracted from specialized publications (*Arbutus*, [103]; *Toddalia*, [104]). All sugar contents are given for fresh fruits.

## 4. Results

### 4.1. Caries morphology in LMK-Pal 5508

**Fig 3:**
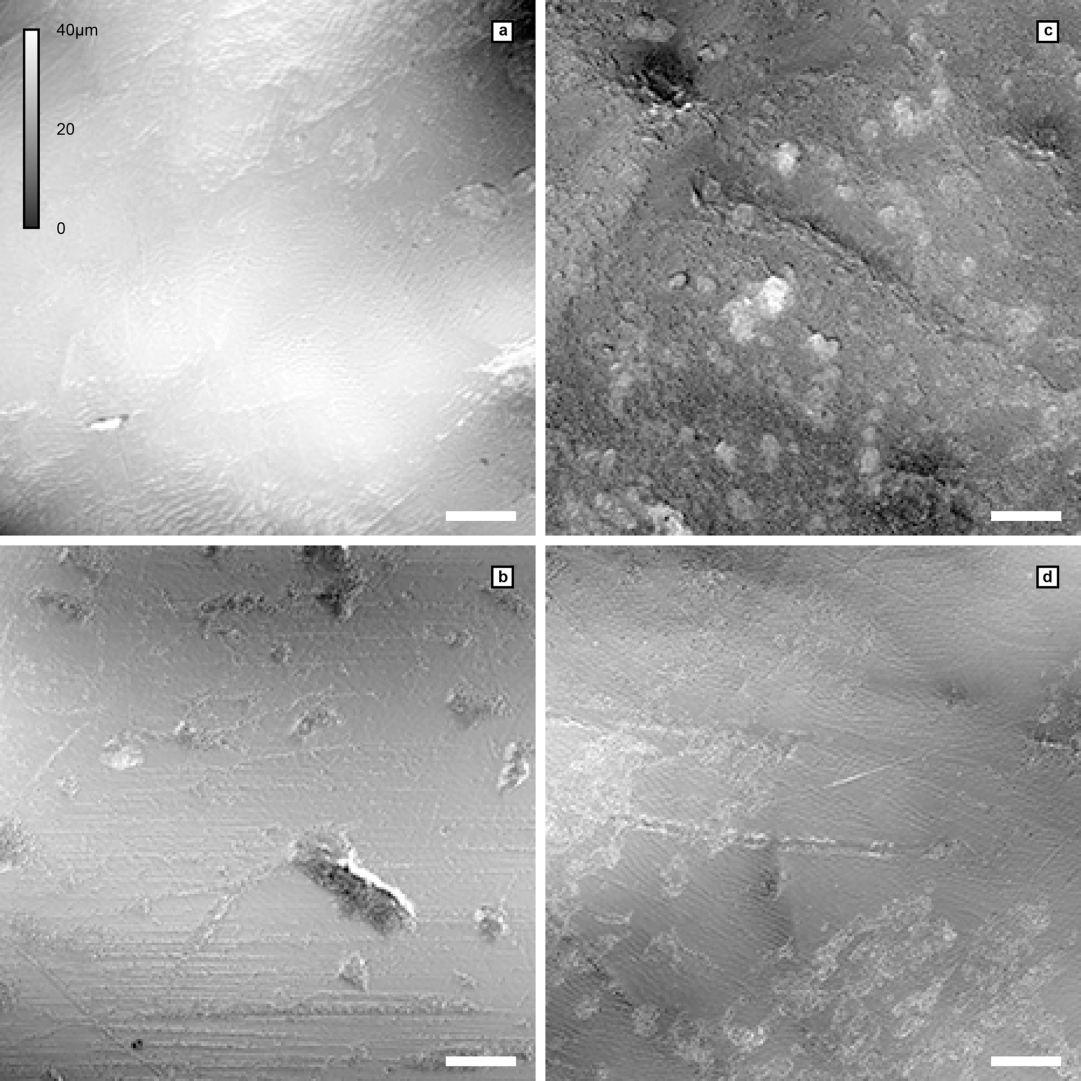
Lasermicroscopic photosimulations showing the wear surface topology in LMK-Pal 5508. **a**, Facet 10 of the left m1, showing inconspicuous scratches and enamel prisms. **b**, Facet 9 of the left m1, showing fine scratches and pits on a polished enamel surface. **c**, Facet 10 of the right m1, showing pits and broad scratches entirely covered by a rough patina. **d**, Facet 9 of the right m1, showing scratches and enamel prisms partially covered by a thin patina. Each photosimulation is 400μm x 400μm; scale bar = 50 μm, magnification = 20x, height differences (0–40μm) in grey-scale.

**Fig 4:**
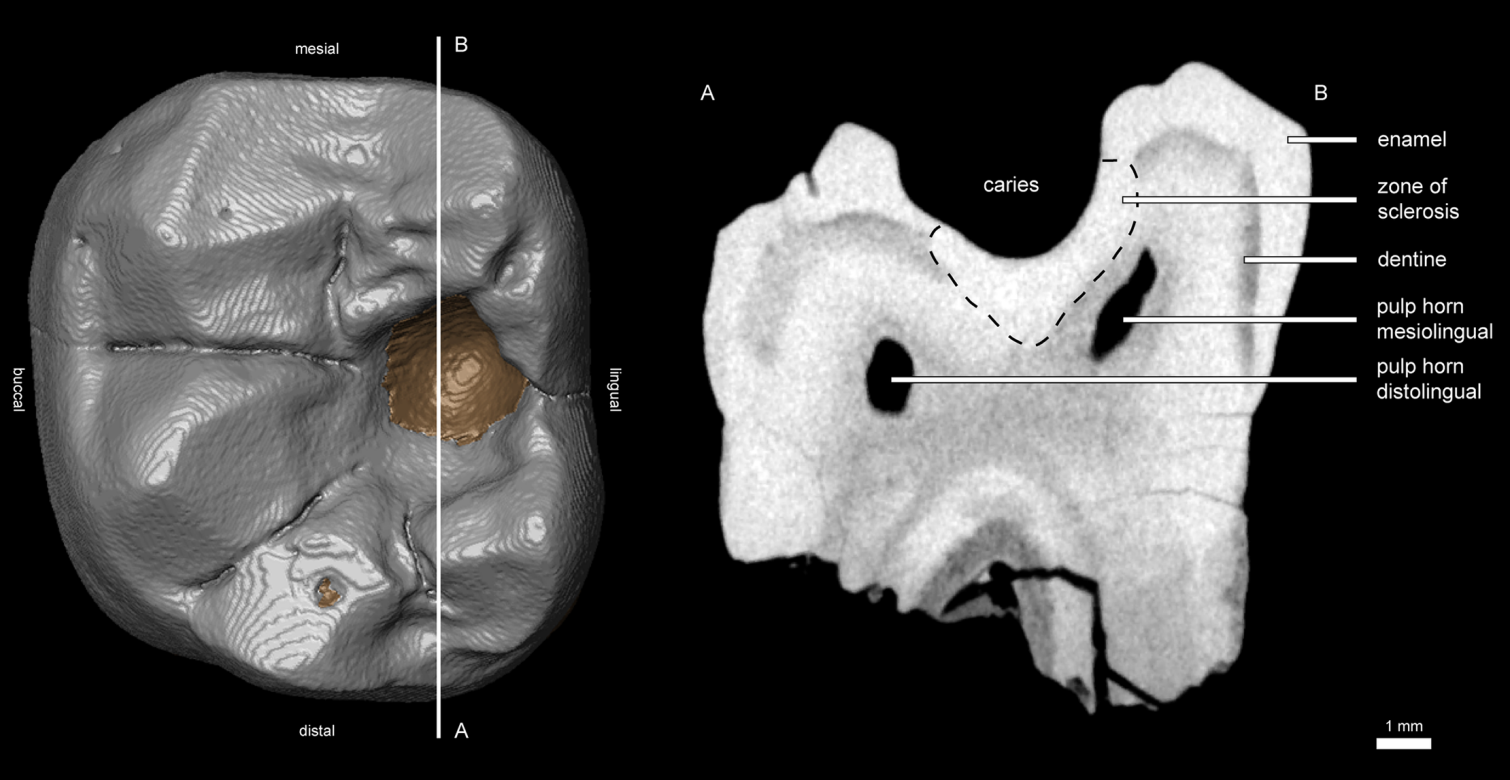
Virtual 3D reconstruction and μCT section of the lower left m1 of *D*. *carinthiacus* (LMK-Pal 5508). Left: μCT-based reconstruction of the left m1 in occlusal view. Enamel cap colored in grey and dentine exposures colored in brown. The mesiodistal section A-B passes through the caries cavity. Right: Corresponding μCT section from A to B showing the caries lesion and internal structures.

**Fig 5:**
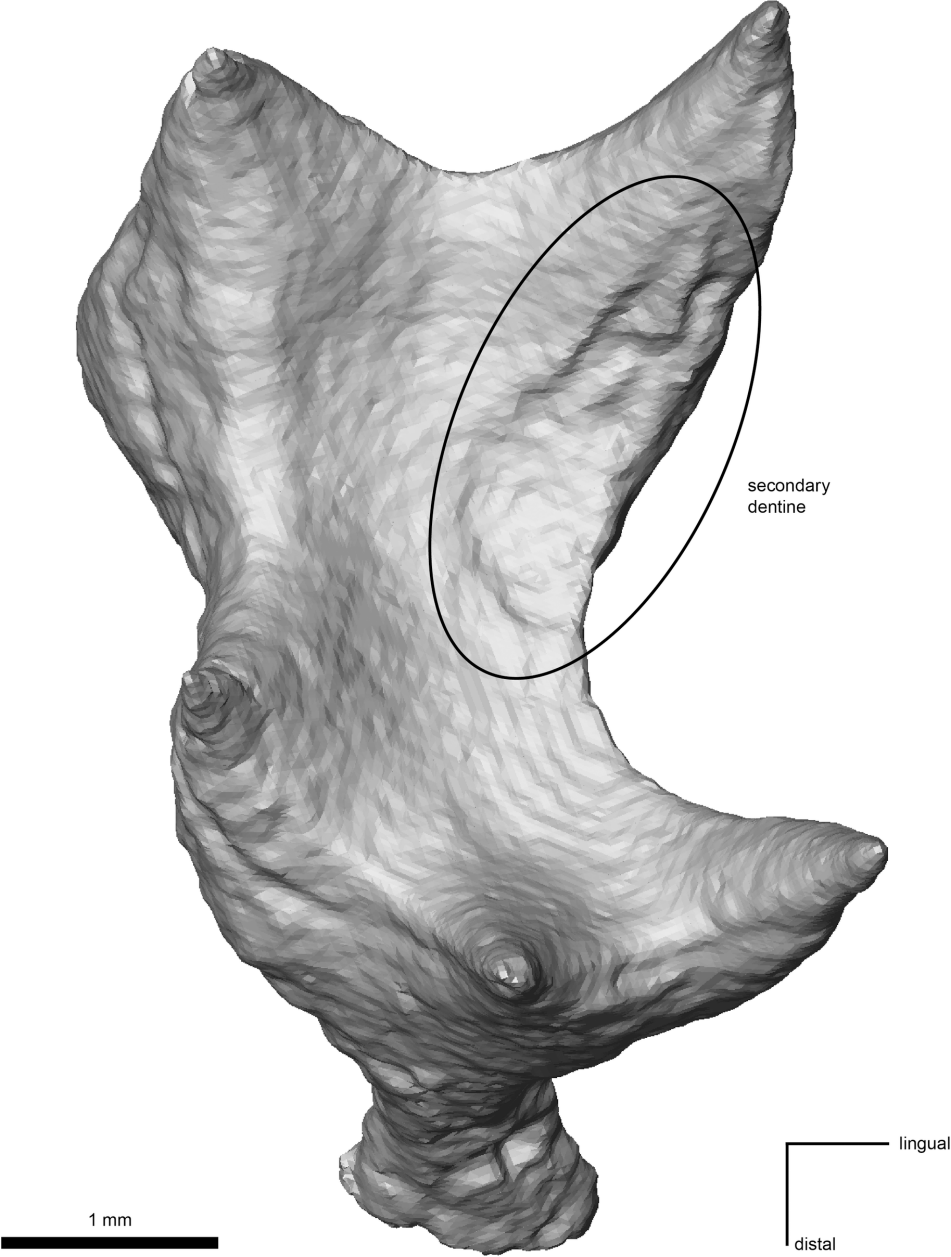
Virtual 3D reconstruction of the left m1 pulp chamber of LMK-Pal 5508 (occlusal view). An irregular depression (circled) distal to the mesiolingual pulp horn indicates accumulation of reparative dentine on the pulp chamber roof below the caries lesion.

**Fig 6:**
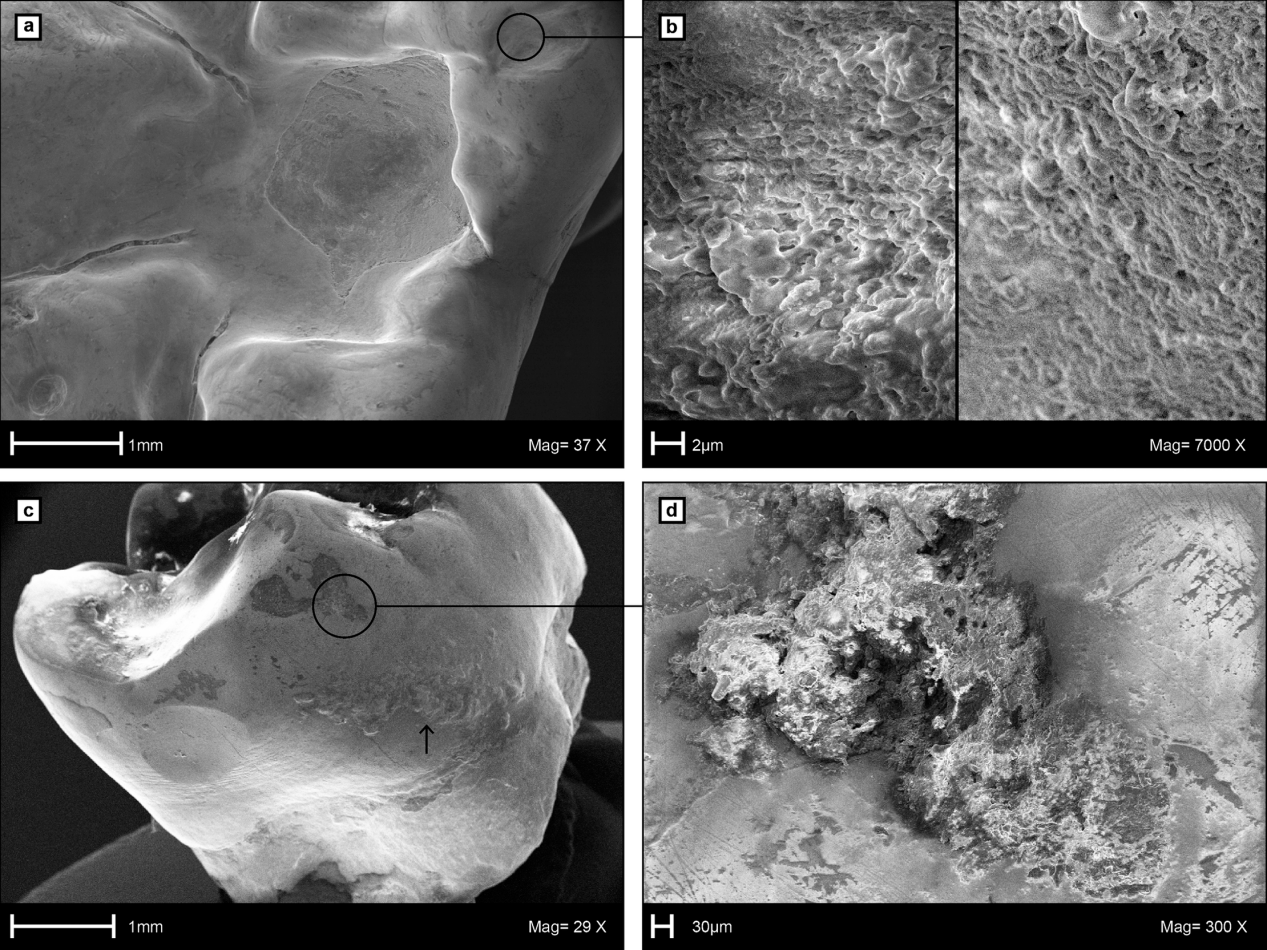
Scanning Electron Microscopy (SEM) images of the caries cavity and calculus on the left m1 of LMK-Pal 5508. **a**, Occlusal view into the caries cavity. **b**, Surface details of occlusal dental calculus close to the caries cavity. **c**, Supra-gingival calculus (above arrow) on the mesiobuccal enamel surface. **d**, Surface details of mesiobuccal calculus and scratch dominated microwear on the enamel surface.

The dental morphology of *D*. *carinthiacus* shows the general dryopithecine characters of narrow and tall-crowned incisors, a buccolingual compressed canine and thinly enameled molars with broad and laterally positioned cusps [77, 105, 106]. It differs from other dryopithecine taxa particularly by its dental dimensions, which are considerably below other dryopithecine specimens [105]. In its first description, Mottl [77] mentioned the severe caries lesion in the left m1 of LMK-Pal 5508. It shows a cavitated pit on the occlusal surface between metaconid and entoconid. The cavitation penetrates the clearly visible EDJ and exposes large parts of dentine. The cavitation through the enamel and the dentine has a maximum diameter of 3.3 mm and 2.4 mm, respectively. The walls are steep and the surface has a smooth etched appearance, as known from acid-producing cariogenic bacteria like *Streptococcus sp*. and *Lactobacillus spp*. Examination with μCT, SEM and stereomicroscopy revealed further morphologies also known from extant human caries lesions:

1. Primary caries. The dental crown of the left m1 shows no fractures and only little occlusal wear. The caries lesion initiated on the intact and largely unworn occlusal enamel surface. This contrasts to secondary caries, which initiates on enamel fractures or dentine exposures due to advanced wear.
2. Unilateral mastication. The left m1 is only slightly worn with a small cuspal dentine exposure on the hypoconulid (Fig 1A). The right m1 shows larger dentine exposures on the buccal cusps (protoconid, hypoconid, hypoconulid; Fig 1B). The other teeth (incisive, canine, p3s and p4s) remain largely unworn, exhibiting only small occlusal and/or interstitial wear facets. None of these teeth exhibit lesions or fractures. The crushing facets in both m1 show little microwear and the wear pattern slightly differs between the left and the right m1 (Fig 3). The wear facets of the right m1 (facet 9 and 10) are largely covered by a thin patina. Where visible, the actual enamel surface is well preserved showing enamel prisms and scratch-dominated microwear; pits are virtually absent here. In contrast, facet 9 of the left m1 shows a polished surface with a large number of fine scratches and a considerable amount of pits (Fig 3B).
3. Plugged dentine. The dentine affected by the caries shows an increased density in the μCT-section (bright zone below the cavity; Fig 4). A hardening of dentine is a typical process when dentine is exposed due to caries or wear. In this zone of sclerosis, mineralized materials plug the dentine tubules and reduce the migration of external substances and microorganisms into the pulp. Calcified bacteria that invaded into the dentine can additionally increase its density. Probably, post mortal, diagenetic processes may also lead to an increase in density by impregnating mineralized waters.
4. Reparative dentine. The pulp chamber shows an irregular depression in the area below the caries cavity (Fig 5), indicating an accumulation of reparative dentine on the pulp chamber roof. Reparative dentine appears early after the caries penetrated the enamel cap down to the dentine [48]. This reparative mechanism protects the pulp chamber from potential contaminations. This means that the pulp of LMK-Pal 5508 was threatened by bacterial infections.
5. Dental calculus. The carious left m1 preserves a macroscopically visible mesiobuccal accretion on the interstitial enamel surface that we interpret as a supra-gingival dental calculus (Fig 6C). No accretions like dental calculus are seen on the other teeth, but they might have been removed during previous preparation work. Under the SEM, further calculus deposits are detected in pits of the occlusal enamel (Fig 6A and 6B) and the mesiobuccal enamel surface (Fig 6C and 6D) of the left m1. The dental calculus shows the characteristic cavernous and spherical deposits that are accumulated by microbiota (Fig 6B and 6D).

### 4.2. Caries frequency in *Pan troglodytes verus*

**Fig 7:**
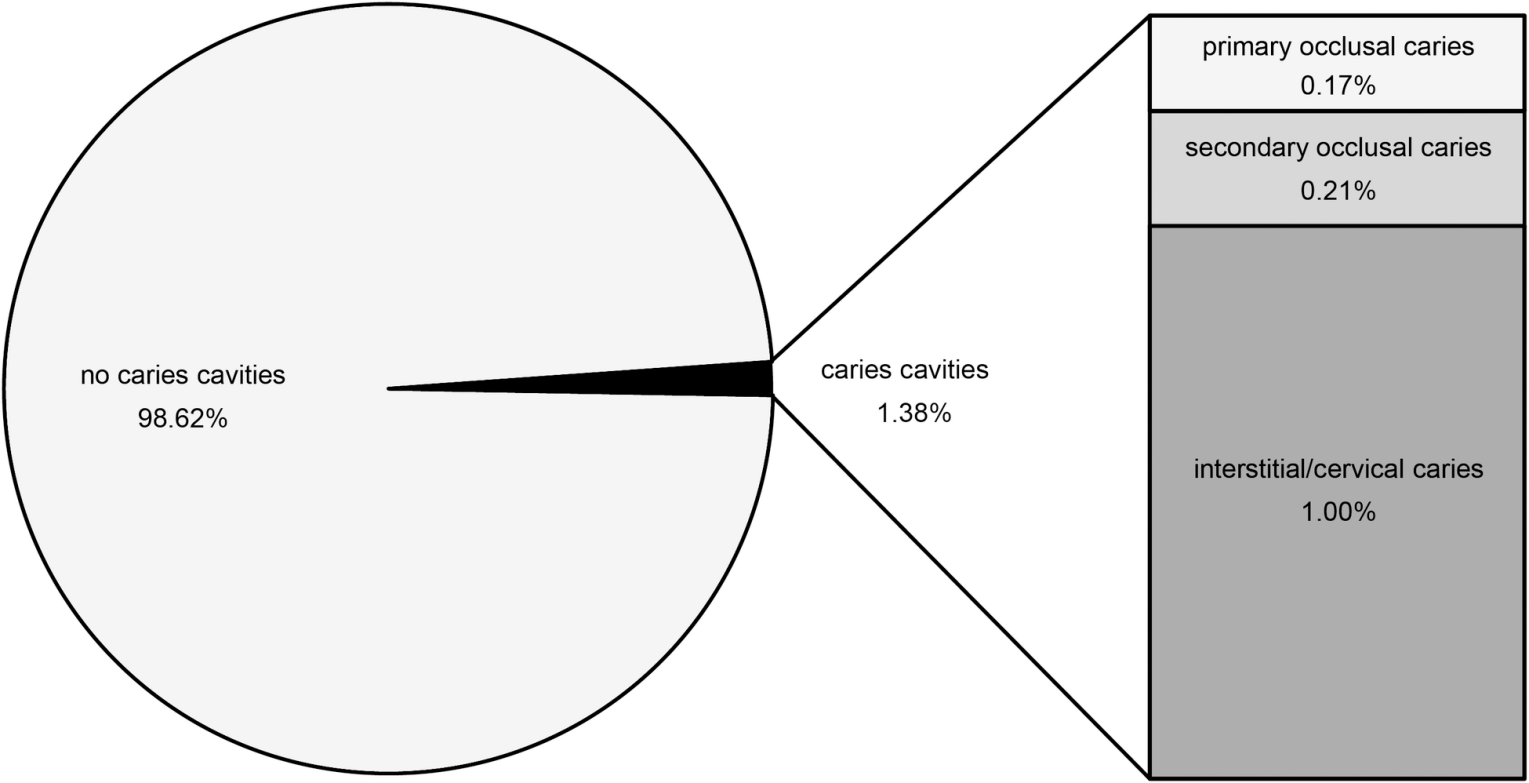
Percentage of non-carious and carious permanent teeth in wild *Pan troglodytes verus* from Liberia (Senckenberg Skull Collection). Within the permanent teeth (n = 2890), interstitial/cervical caries occurs in 1.00% (n = 29), secondary occlusal caries in 0.21% (n = 6) and primary occlusal caries in 0.17% (n = 5).

Our examination of 311 chimpanzees revealed 16 individuals with carious teeth. From a total of n = 2890 permanent teeth and n = 369 deciduous teeth, caries cavities occur in 1.38% (n = 40) of the permanent teeth (Fig 7) and are absent in the deciduous ones. With 0.17% (n = 5) of total permanent teeth, the primary occlusal caries is very rare, followed by the frequency of secondary occlusal caries (0.21%; n = 6). With n = 29 teeth, cervical/interstitial caries is more frequent, constituting 1% of the total permanent teeth (Fig 7).

**Fig 8:**
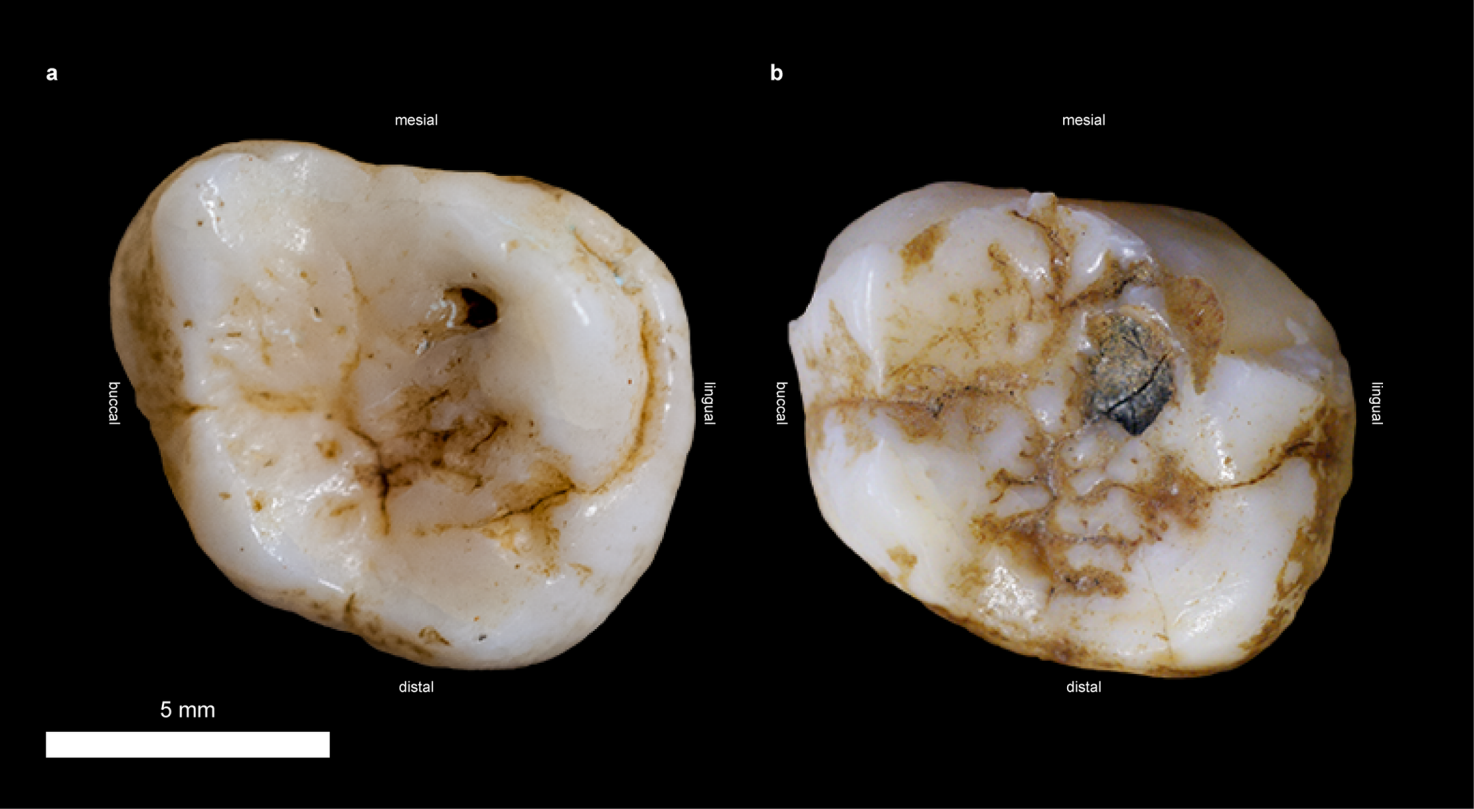
Two exemplary cases of primary occlusal caries in *Pan troglodytes verus*. **a**, Small caries pit in a right M3 (Specimen 34-SMF/PA/PC 34). **b**, Broad but shallow caries pit in a right M2 (Specimen 246-SMF/PA/PC 246). Teeth are graphically isolated from the maxilla.

The most severe caries lesions, featuring deep cavities with large diameters of several millimeters, belong to the secondary occlusal type and the interstitial/cervical type. The primary occlusal caries is less pronounced, featuring only shallow or tiny pits of less than one millimeter in diameter (Fig 8).

### 4.3. Potential sources of cariogenic sugars

**Fig 9:**
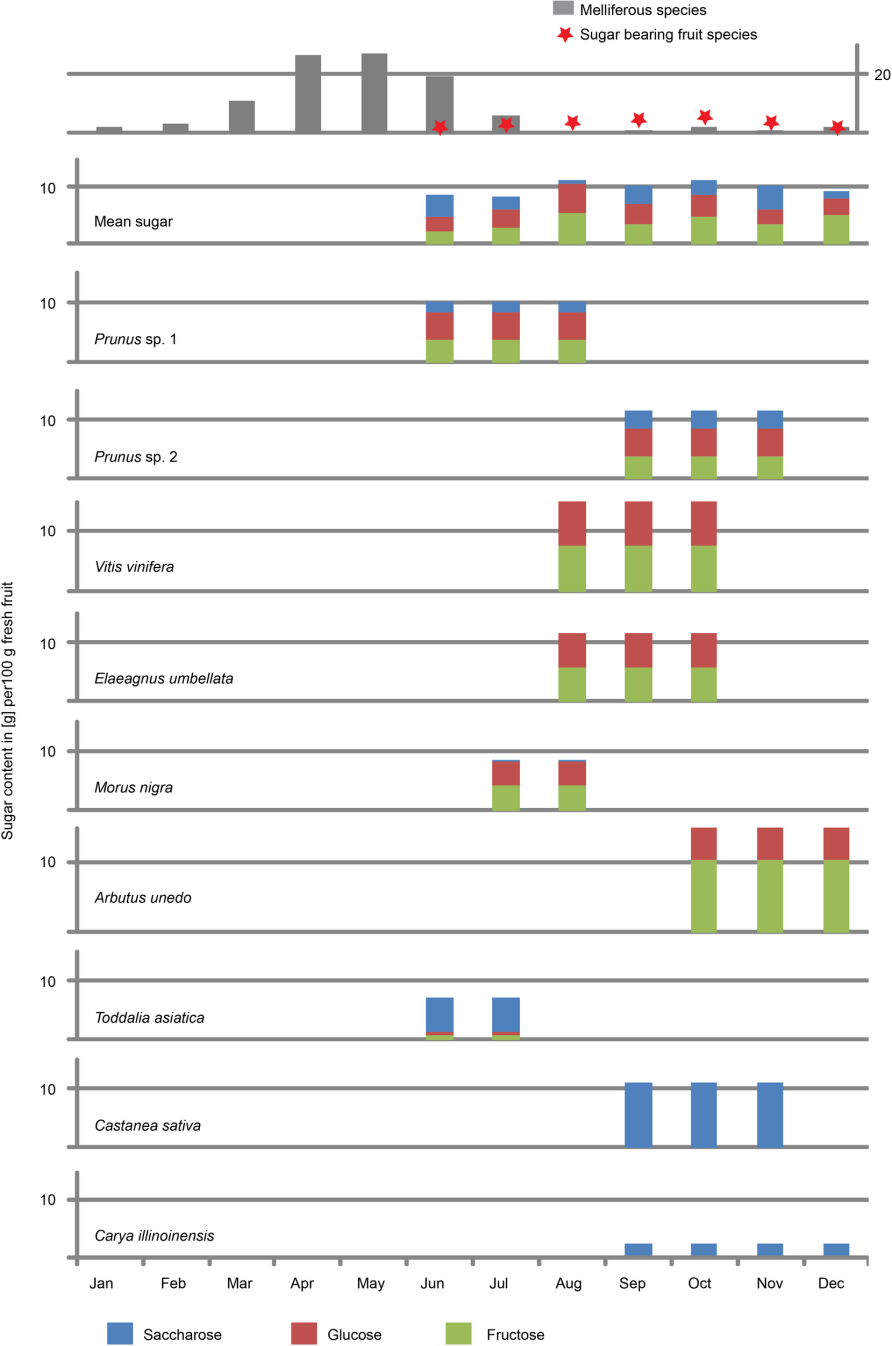
Soluble non-structural carbohydrate content (mono- and disaccharides) in ripe fruits from nearest living relatives of palynological taxa identified in phosphorite nodules from St. Stefan [78, 96–99] and their corresponding ripening periods. *Prunus* sp. 1 refers to early ripening (*P*. *avium*, *P*. *cerasus*, *P*. *armeniaca*) and *Prunus* sp. 2 to late ripening species (*P*. *spinosa*, *P*. *domestica*, *P*. *syriaca*). The average sugar content of fruits available per month is displayed in the second diagram from above. The top diagram represents the number of melliferous tree and climber species per flowering month (S2 Table) and the number of sugar containing fruit species per ripening month.

Searching for potential food plants in published floral lists [78, 98, 99] revealed at least nine palynological taxa from various plant orders (Rosales, Fagales, Vitales, Ericales, and Sapindales), which produced fruits that contained considerable amounts of mono- and/or disaccharides. We identified the following taxa (Fig 9): *Prunus* sp. 1 and 2 (e.g. cherry, apricot, plum, peach, almond; Rosales), *Vitis* sp. (grape; Vitales), *Elaeagnus* sp. (oleaster/Russian olive; Rosales), *Morus* cf. *nigra* (black mulberry; Rosales), *Arbutus* sp. (strawberry tree, Ericales), *Castanea* sp. (chestnut; Fagales), *Carya* sp. (hickory; Fagales), *Toddalia* sp. (orange climber; Sapindales). Especially members of Rosales, Vitales, and Ericales are rich in monosaccharides and their content of fructose reach, or exceed 50% of all non-structural carbohydrates, whereas fruits of Fagales and Sapindales are dominated by disaccharides (Fig 9).

All nine fruits ripe on a seasonal basis and estimations of their fruiting time suggest availability for at least seven months (June-December; Fig 9). With five to six species of available sweet fruits, the most diverse season is autumn (September-October), whereas mean sugar and monosaccharide contents remain high in all fruiting months. The relative proportion of fructose is highest during December (56% of total sugar), caused by the fructose dominated strawberry tree (*Arbutus unedo*).

#### Availability of honey

The nutritional availability of honey for extinct hominids cannot be directly documented. However, if the diversity and abundance of melliferous plants are high, honey will be available and will probably be consumed by hominids [107]. The taxonomic resolution of the palynological record is too low to estimate the importance of melliferous herbs. However, the Lavanttal palynoflora provides at least 46 potential melliferous tree and climber taxa (see S2 Table), which notably include the most abundant arboreal elements like *Quercus* ssp., *Carya* sp., *Fagus* sp., *Castanea* sp., *Ulmus* sp., and *Pinus* ssp. comprising about 50% of recorded pollen grains [78]. We therefore suggest that honey might have been nutritionally available to *D*. *carinthiacus*. Furthermore, the season of honey availability was estimated based on the flowering months of documented melliferous taxa (see S2 Table). The flowering periods center between early spring to early summer (March–July; Fig 9), with maximum diversity of blooming melliferous species and therefore honey availability during April and May (26 and 27 taxa respectively).

## 5. Discussion

### 5.1. Etiology of caries for *D*. *carinthiacus*

In general, caries is a multifactorial disease, which cannot be narrowed down to a single cause. Dietary preferences, physiological differences due to age and sex, or individual susceptibility to plaque and caries formation (e.g. tooth surface morphology, enamel quality, oral flora and other predispositions) might be responsible for carious teeth.

#### Age, dental wear and diet

In wild great apes, severe caries mostly occurs interstitial/cervical or in teeth where dentine is exposed due to heavy wear or fractures (Fig 7 and Miles and Grigson [53]). These formation types of ‘interstitial/cervical’ and ‘secondary occlusal’ are particularly prevalent in old individuals [53, 74]. This pattern is consistent with our dataset of Liberian chimpanzees (S1 Table), which shows intense caries only in permanent and often heavily worn teeth, while juveniles are completely unaffected. The increasing incidence and severity of caries with age can partially be explained by the progressive course of the cavitation process in a steady state of risk, as it may apply to animals and Pleistocene hominins. Further, the increasingly worn teeth expose a greater area of dentine. Compared to enamel, dentine shows a lower degree of mineralization which accompanies with a higher susceptibility to plaque and caries in worn teeth. In turn, intense dental wear might catch up the cavitation process and counteract against it [54]. The major cause of caries in *D*. *carinthiacus* grounds on other, mainly diet specific factors. Contrasting to the prevailing cases of caries in extant wild apes, LMK-Pal 5508 is a young adult (largely unworn teeth, at least m2 erupted as evident from the distal interstitial wear facet on both m1). Its carious left m1 is largely unworn and shows no fractures that could have facilitate caries initiation. This infers that the caries affected the intact occlusal enamel and hence, can be described as primary caries [53]. Primary caries is a common disease of humans in recent industrial societies, but rather unusual for wild animals. This is confirmed by our results on caries frequencies in extant chimpanzees, where only 0.17% of investigated teeth show primary caries lesions (Fig 7). Furthermore, observed primary caries in *Pan troglodytes verus* (Fig 8) is found to be less pronounced compared to *D*. *carinthiacus*, which suggests less severe cariogenic effects for affected individuals of chimpanzees.

The growing incidence of primary caries in human history clearly correlates with the increased consumption of cocked starch and sugar-rich products [53, 62]. This may indicate that the deep primary caries in LMK-Pal 5508 has a dietary etiology, as well. Sugar-rich fruits could have been a possible natural source. Inferred from caries formation in humans [50], it can be assumed that the advanced cavitation in LMK-Pal 5508 needed several years to form. Accordingly, we assume a repetitive consumption of carbohydrates for this individual.

Certainly, irrevocable inferences about the dietary niche of *D*. *carinthicacus* are hampered by the single specimen known from this species. However, the overall dental morphology of *D*. *carinthiacus* coincides with our assumption, showing the characteristic frugivorous configuration of small teeth with thin enamel [106] and broad cusps. Tentatively, the largely scratch dominated microwear-pattern in *D*. *carinthiacus* infers a rather soft diet that included some leaves (Fig 3). This might be supported by the presence of patina layers, which are common in leaf-eating monkeys like *Colobus* [108]. Their patinas are interpreted as a mechanism to protect the enamel from lateral abrasion [108].

#### Sex-related

Although questionable, the single known individual of *D*. *carinthiacus* is discussed to be a female *D*. *fontani*, due to a similar morphology, but its smaller size [77, 109]. Female primates tend to have a higher frequency of caries than males, due to physiological and behavioral reasons [62, 74]. Medical studies on humans indicate that hormonal fluctuations during menstruation and pregnancy influence the chemical composition and amount of saliva [62, 110]. This may lead to xerostomy and a low pH, which promote the formation of caries. An additional, more controversial factor is the potential calcium deficiency that might occur during pregnancy and lactation [110, 111]. Beside such physiological factors, also dietary differences between sexes might influence the incidence of caries. Stoner [74] documented a higher frequency of caries and other dental pathologies in adult female orangutan. She explains this by observations that females eat more fruits compared to males, which concentrate more on bark and ground resources.

### 5.2. Dietary inferences for *D*. *carinthiacus*

Apart from honey, the most cariogenic diets available in the environment of *D*. *carinthicus* were ripe fruits. The palynological record of St. Stefan documents at least nine plants that may have served as source of consumable, sugar-rich fruits (Fig 9). The diversity of these fruits was highest during the autumn and mean sugar content was high all the fruiting season (seven month from June-December). Thus, the mixed mesophytic forest of the early Sarmatian Lavanttal Basin, with its seasonal fruiting trees and honey, provided diverse sugar resources for up to nine or ten months a year, from late spring to early winter. Sugar-rich fruits have been unavailable in late winter and spring times and honey was rare from late summer to late winter. Interestingly, fructose was the dominant sugar in fruits only in December, suggesting enhanced lipogenesis just before the starvation season in late winter. Given the northern latitude of the Lavanttal, we think that especially the late winter months (January, February), which received considerably less insolation during short days, held risk of starvation. This would demand catabolism of fat reserves (as described for the tropical *Pongo pygmaeus* by Vogel, Knott [112]). Foraging on fructose-rich fruits of the genera *Prunus*, *Vitis*, *Elaeagnus*, *Morus*, and *Arbutus* could has generated these fat reserves. Especially the latter taxon may potentially represent a key-species, since it was responsible for the fructose surplus in December, just before the inferred starvation period.

### 5.3. Food quality, foraging strategies and metabolism

Both morphologic (enamel thickness, cusp morphology, enamel microstructure, caries lesion) and environmental data characterize *D*. *carinthiacus* as a frugivore, preferentially feeding on high-quality, sugar-rich fruits, similar to chimpanzees [113, 114]. As the fleshy fruits of the identified taxa are low in fiber content (1–9% [102]), we further expect seasonally low fiber values in the diet of *D*. *carinthiacus*, contrasting to the frugivorous, though fibrous diet of chimpanzees [115]. According, *D*. *carinthiacus* indicates that foraging on high quality foods was already present in early hominids. Moreover, this strategy combined with a change in purine metabolism might have been a first step to cope with the increasing seasonality during the Miocene.

Temporal and spatial variation in the food supply requires a high flexibility in behavior, ranging and diet of primates [116]. In such habitats, larger-sized apes like *D*. *carinthiacus* have several advantages compared to small-bodied primates. A large gut allow them to process less digestible diets like leaves [116]. In times of food scarcity, large primates show a larger range to track their resources. Further, their larger body-fat stores enable them to withstand periods of starvation [116].

In contrast to specialized folivores, *D*. *carinthiacus* showed a more flexible diet dominated by fruits. Its generalized tooth morphology facilitated the processing of these high-quality foods, though its thin enamel made it prone to caries disease.

**Fig 10:**
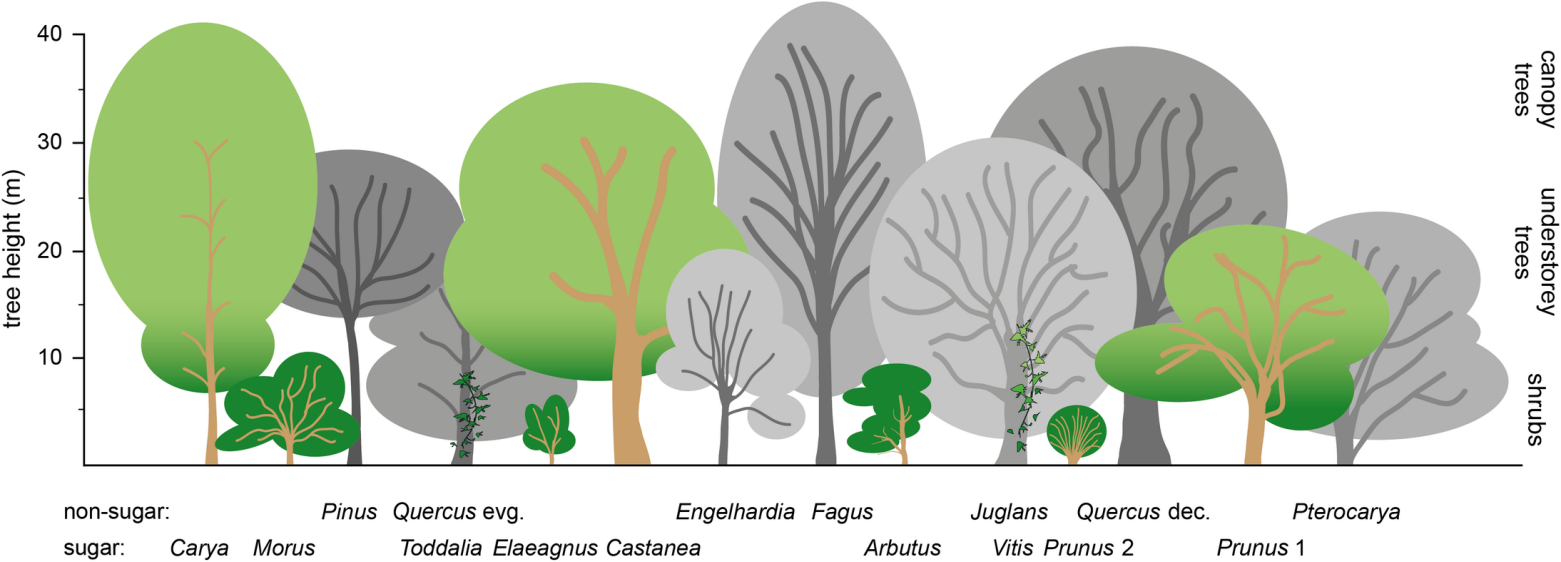
Supposed structure of the early Sarmatian Lavanttal lowland forest, showing layers of shrubs, understorey, and canopy trees. Trees that dominate the pollen spectra [78] are shown in grey scales. Trees, shrubs, and vines producing sugar-containing fruits are colored. Note that the dominant source of sugar-rich fruits is the shrub layer.

As revealed by palynology, available fruits derived from trees and climbers, suggesting further a canopy feeding adaptation. Most feeding trees (except *Castanea* and *Carya*) can be regarded as small- to medium sized (5 to 12 m high), with fruit concentration on small terminal branches (Fig 10). We therefore assume that *D*. *carinthiacus* developed skills for terminal branch eating, such as a form of suspensory posture. Known from several extant platyrrhines, cercopithecids and all extant hominoids, hindlimb- and forelimb-suspensory is an ideal adaption for a better weight distribution on small supports and well suited to collect food items below terminal branches [117].

As a major fall-back food, the extant frugivorous primates of the tropics largely depend on young leaves [13]. However, in the higher latitudes of St. Stefan, the production of young leaves might not bridge the time of food scarcity during late winter. If this is the case, other strategies like the switch to low-quality foods or the early build-up of fat reserves were required [118].

**Fig 11:**
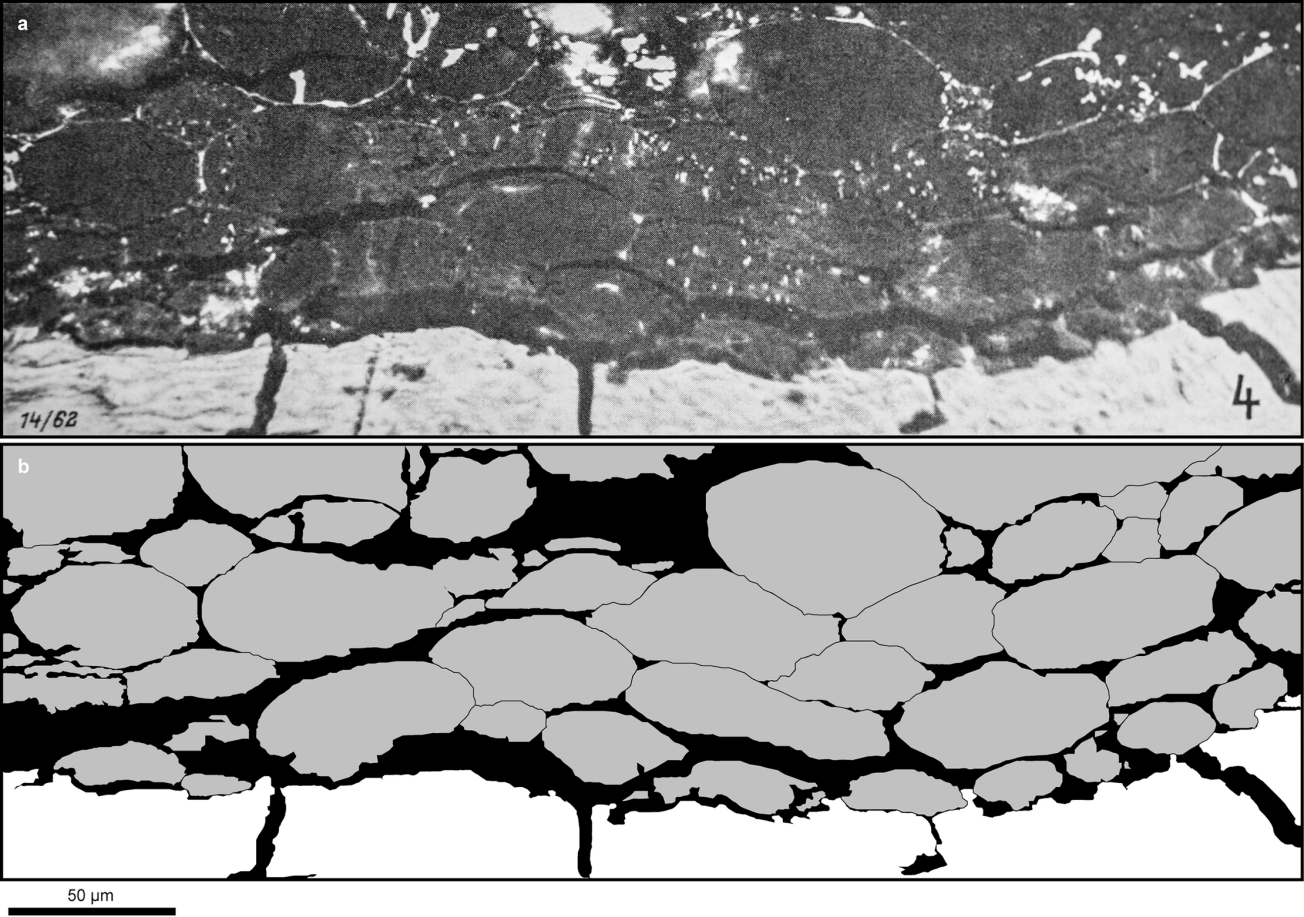
Potential adipose tissue (dark, resinite-like cell bond with cell sizes up to 60 μm) from close to the *Oreopithecus*-skeleton of Baccinello (Italy). A: Polished thick section, under oil immersion and 270x magnification. Reproduction of plate 16, Fig 4 published by Teichmüller [119]. B: Simplified drawing of the thick section above, highlighting the cell structure (in grey).

Due to its frugivory and body size, as well as its carbohydrate-rich habitat with high predictability of the fruiting season, *D*. *carinthiacus* is predestined to rely on the early storage of fat reserves to endure periods of starvation. The hominid fructose and uric acid metabolism stimulate lipogenesis and lead to elevated body fat accumulation, relative to iso-caloric fructose-poor diet (see chapter 1.3.). We have shown that the potential food of *D*. *carinthiacus* was rich in fructose, especially in early winter month, which is related to the typically Mediterranean tree *Arbutus* and others. This led us speculate that *D*. *carinthiacus* and potentially other European hominids may have seasonally accumulated substantial adipose tissue. Identification of large adipocyte-like structures in preserved soft-tissue from the Late Miocene ape *Oreopithecus bamboli* (Fig 11) [119] corroborate this hypothesis. Their large size (up to 60 μm) and spherical shape would characterize them as white adipose cells (lipids), responsible for energy storage, in contrast to the smaller and polygonal brown adipose cells, which are involved in thermogenesis [120].

## 6. Conclusions

The type of *D*. *carinthiacus* (LMK-Pal 5508) dates to the Early Sarmatian (Middle Miocene) at ~12.5 Ma, thus being the earliest known representative of the dryopithecine clade. Furthermore, this specimen shows the earliest record of primary dental caries and calculus in a hominid. The caries lesion in *D*. *carinthiacus* is indicated on features known from severe dental caries in humans: (1) The cavitation has a smooth surface and steep walls due to erosion by acids; (2) Reparative dentine at the roof of the pulp chamber; (3) Probably plugged dentine, due to caries undermining the enamel; (4) Association with dental calculus at different sites on the enamel surface; (5) Unilateral usage of the healthy, right tooth row indicates that LMK-Pal 5508 may have suffered on toothache.

The main culprit of this caries, the frequent consumption of highly cariogenic food (sugar-rich diets like fruits or honey), is indicated by the presence of deep primary caries that initiated on the intact enamel surface. This caries morphology contrasts to the rare and weakly developed primary caries in *Pan troglodytes verus* and the common interstitial/cervical and secondary caries that initiates by lesions or dental wear. According, we infer a highly frugivorous diet for *D*. *carinthiacus*, which likely exceeds the frugivory of extant chimpanzees.

The palynological record of St. Stefan indicates a seasonally rich fructiferous flora that would have allowed the reliance on a highly frugivorous diet during nine or ten months a year. Such a nutrition corresponds to the uricase-hypothesis of Johnson and Andrews [6], which discusses the hominid specific development of a fructose-based metabolism to build up fat reserves for periods of starvation. This adaption is characterized by the loss of a functional uricase enzyme during the Middle Miocene [121] and likely evolved in the seasonally driven habitats of Eurasia [6]. Beside *D*. *carinthiacus*, this adaption might have been present in many other European hominids as documented by the potential white adipose tissue of *Oreopithecus bamboli*. Further, the quality of the diet available to *D*. *carinthiacus* was superior (high carbohydrate, low fiber) to that of living great apes, suggesting that the model of step-wise increase in dietary quality [115] during hominid evolution has to be modified.

## Supporting information

S1 Table Counting of caries cavity types in permanent and deciduous teeth of *Pan troglodytes verus* SCHWARZ 1934, from the Senckenberg skull collection of Liberian chimpanzees. (DOCX)

S2 Table Potential melliferous plants of the Lavanttal flora. (DOCX)
