# Supplementary material for: Earliest evidence of caries lesion in hominids reveal sugar-rich diet for a Middle Miocene dryopithecine from Europe
Source: PLoS One. 2018 Aug 30;13(8):e0203307. doi: 10.1371/journal.pone.0203307 (PMC6117023; doi:10.1371/journal.pone.0203307)
Supplement: S1 Table — (DOCX) [file pone.0203307.s001.docx]

**S1 Table. Counting of caries cavity types in permanent and deciduous teeth of *Pan troglodytes verus* SCHWARZ 1934, from the Senckenberg skull collection of Liberian chimpanzees.**

| Catalogue number | n permanent teeth | n deciduous teeth | n  primary  occlusal caries | n  secondary  occlusal caries | n  interstitial/  cervical  caries |
| --- | --- | --- | --- | --- | --- |
|  | (N= 2890) | (N= 369) | (N= 5) | (N= 6) | (N= 29) |
| 1-SMF/PA/PC 1 | 6 | 0 | 0 | 0 | 0 |
| 2-SMF/PA/PC 2 | 5 | 0 | 0 | 0 | 0 |
| 3-SMF/PA/PC 3 | 0 | 0 | 0 | 0 | 0 |
| 4-SMF/PA/PC 4 | 6 | 0 | 0 | 0 | 0 |
| 5-SMF/PA/PC 5 | 6 | 0 | 0 | 0 | 0 |
| 6-SMF/PA/PC 6 | 0 | 0 | 0 | 0 | 0 |
| 7-SMF/PA/PC 7 | 2 | 0 | 0 | 0 | 0 |
| 8-SMF/PA/PC 8 | 5 | 0 | 0 | 0 | 0 |
| 9-SMF/PA/PC 9 | 5 | 0 | 0 | 0 | 0 |
| 10-SMF/PA/PC 10 | 5 | 0 | 0 | 0 | 0 |
| 12-SMF/PA/PC 12 | 4 | 0 | 0 | 0 | 0 |
| 13-SMF/PA/PC 13 | 4 | 0 | 0 | 0 | 0 |
| 14-SMF/PA/PC 14 | 8 | 0 | 0 | 0 | 0 |
| 15-SMF/PA/PC 15 | 0 | 0 | 0 | 0 | 0 |
| 16-SMF/PA/PC 16 | 9 | 0 | 0 | 0 | 0 |
| 17-SMF/PA/PC 17 | 6 | 0 | 0 | 0 | 0 |
| 19-SMF/PA/PC 19 | 4 | 0 | 0 | 0 | 0 |
| 21-SMF/PA/PC 21 | 2 | 13 | 0 | 0 | 0 |
| 22-SMF/PA/PC 22 | 0 | 0 | 0 | 0 | 0 |
| 23-SMF/PA/PC 23 | 7 | 0 | 0 | 0 | 0 |
| 24-SMF/PA/PC 24 | 8 | 0 | 0 | 0 | 0 |
| 25-SMF/PA/PC 25 | 0 | 0 | 0 | 0 | 0 |
| 26-SMF/PA/PC 26 | 3 | 0 | 0 | 0 | 0 |
| 27-SMF/PA/PC 27 | 1 | 0 | 0 | 0 | 0 |
| 29-SMF/PA/PC 29 | 6 | 0 | 0 | 0 | 0 |
| 30-SMF/PA/PC 30 | 0 | 0 | 0 | 0 | 0 |
| 31-SMF/PA/PC 31 | 5 | 0 | 0 | 0 | 0 |
| 32-SMF/PA/PC 32 | 8 | 0 | 0 | 0 | 0 |
| 33-SMF/PA/PC 33 | 6 | 0 | 0 | 0 | 0 |
| 34-SMF/PA/PC 34 | 8 | 0 | 1 | 0 | 0 |
| 35-SMF/PA/PC 35 | 8 | 0 | 0 | 0 | 0 |
| 36-SMF/PA/PC 36 | 5 | 0 | 0 | 0 | 0 |
| 38-SMF/PA/PC 38 | 0 | 0 | 0 | 0 | 0 |
| 39-SMF/PA/PC 39 | 10 | 0 | 0 | 0 | 0 |
| 40-SMF/PA/PC 40 | 7 | 0 | 0 | 0 | 0 |
| 41-SMF/PA/PC 41 | 5 | 0 | 0 | 0 | 0 |
| 42-SMF/PA/PC 42 | 0 | 0 | 0 | 0 | 0 |
| 43-SMF/PA/PC 43 | 0 | 0 | 0 | 0 | 0 |
| 44-SMF/PA/PC 44 | 2 | 0 | 0 | 0 | 0 |
| 45-SMF/PA/PC 45 | 0 | 0 | 0 | 0 | 0 |
| 47-SMF/PA/PC 47 | 2 | 0 | 0 | 0 | 0 |
| 49-SMF/PA/PC 49 | 5 | 0 | 0 | 0 | 0 |
| 51-SMF/PA/PC 51 | 26 | 0 | 0 | 0 | 0 |
| 52-SMF/PA/PC 52 | 7 | 0 | 0 | 0 | 0 |
| 53-SMF/PA/PC 53 | 7 | 0 | 0 | 0 | 0 |
| 55-SMF/PA/PC 55 | 7 | 0 | 0 | 0 | 0 |
| 56-SMF/PA/PC 56 | 0 | 10 | 0 | 0 | 0 |
| 57-SMF/PA/PC 57 | 4 | 16 | 0 | 0 | 0 |
| 58-SMF/PA/PC 58 | 3 | 0 | 0 | 0 | 0 |
| 59-SMF/PA/PC 59 | 3 | 0 | 0 | 0 | 0 |
| 60-SMF/PA/PC 60 | 1 | 0 | 0 | 0 | 0 |
| 61-SMF/PA/PC 61 | 4 | 0 | 0 | 0 | 0 |
| 62-SMF/PA/PC 62 | 0 | 16 | 0 | 0 | 0 |
| 63-SMF/PA/PC 63 | 1 | 0 | 0 | 0 | 0 |
| 64-SMF/PA/PC 64 | 4 | 0 | 0 | 0 | 0 |
| 65-SMF/PA/PC 65 | 2 | 0 | 0 | 0 | 0 |
| 66-SMF/PA/PC 66 | 7 | 0 | 0 | 0 | 0 |
| 67-SMF/PA/PC 67 | 0 | 0 | 0 | 0 | 0 |
| 68-SMF/PA/PC 68 | 5 | 0 | 0 | 0 | 0 |
| 69-SMF/PA/PC 69 | 7 | 0 | 0 | 0 | 0 |
| 70-SMF/PA/PC 70 | 6 | 0 | 0 | 0 | 0 |
| 71-SMF/PA/PC 71 | 2 | 0 | 0 | 0 | 0 |
| 72-SMF/PA/PC 72 | 1 | 0 | 0 | 0 | 0 |
| 73-SMF/PA/PC 73 | 8 | 0 | 0 | 0 | 0 |
| 74-SMF/PA/PC 74 | 3 | 0 | 0 | 0 | 0 |
| 75-SMF/PA/PC 75 | 9 | 0 | 0 | 0 | 0 |
| 76-SMF/PA/PC 76 | 4 | 0 | 0 | 0 | 0 |
| 77-SMF/PA/PC 77 | 2 | 0 | 0 | 0 | 0 |
| 78-SMF/PA/PC 78 | 3 | 16 | 0 | 0 | 0 |
| 79-SMF/PA/PC 79 | 1 | 0 | 0 | 0 | 0 |
| 80-SMF/PA/PC 80 | 0 | 0 | 0 | 0 | 0 |
| 81-SMF/PA/PC 81 | 4 | 0 | 0 | 0 | 0 |
| 82-SMF/PA/PC 82 | 2 | 0 | 0 | 0 | 0 |
| 84-SMF/PA/PC 84 | 5 | 0 | 0 | 0 | 0 |
| 85-SMF/PA/PC 85 | 4 | 20 | 0 | 0 | 0 |
| 86-SMF/PA/PC 86 | 3 | 0 | 0 | 0 | 0 |
| 87-SMF/PA/PC 87 | 2 | 0 | 0 | 0 | 0 |
| 88-SMF/PA/PC 88 | 0 | 1 | 0 | 0 | 0 |
| 89-SMF/PA/PC 89 | 6 | 0 | 0 | 0 | 0 |
| 90-SMF/PA/PC 90 | 11 | 0 | 0 | 0 | 0 |
| 91-SMF/PA/PC 91 | 6 | 0 | 0 | 0 | 0 |
| 93-SMF/PA/PC 93 | 7 | 0 | 0 | 0 | 0 |
| 94-SMF/PA/PC 94 | 0 | 0 | 0 | 0 | 0 |
| 95-SMF/PA/PC 95 | 5 | 0 | 0 | 0 | 0 |
| 96-SMF/PA/PC 96 | 7 | 0 | 0 | 0 | 0 |
| 98-SMF/PA/PC 98 | 0 | 6 | 0 | 0 | 0 |
| 99-SMF/PA/PC 99 | 24 | 0 | 0 | 0 | 0 |
| 100-SMF/PA/PC 100 | 19 | 0 | 0 | 0 | 0 |
| 101-SMF/PA/PC 101 | 3 | 0 | 0 | 0 | 0 |
| 102-SMF/PA/PC 102 | 4 | 0 | 0 | 0 | 0 |
| 103-SMF/PA/PC 103 | 0 | 0 | 0 | 0 | 0 |
| 104-SMF/PA/PC 104 | 5 | 0 | 0 | 0 | 0 |
| 107-SMF/PA/PC 107 | 7 | 0 | 0 | 0 | 0 |
| 108-SMF/PA/PC 108 | 7 | 0 | 0 | 0 | 0 |
| 109-SMF/PA/PC 109 | 5 | 0 | 0 | 0 | 0 |
| 110-SMF/PA/PC 110 | 5 | 0 | 0 | 0 | 0 |
| 111-SMF/PA/PC 111 | 5 | 0 | 0 | 0 | 0 |
| 112-SMF/PA/PC 112 | 2 | 0 | 0 | 0 | 0 |
| 113-SMF/PA/PC 113 | 6 | 0 | 0 | 0 | 0 |
| 114-SMF/PA/PC 114 | 11 | 0 | 0 | 0 | 0 |
| 115-SMF/PA/PC 115 | 5 | 0 | 0 | 0 | 0 |
| 116-SMF/PA/PC 116 | 6 | 0 | 0 | 0 | 0 |
| 117-SMF/PA/PC 117 | 7 | 0 | 0 | 0 | 0 |
| 118-SMF/PA/PC 118 | 3 | 0 | 0 | 0 | 0 |
| 120-SMF/PA/PC 120 | 0 | 0 | 0 | 0 | 0 |
| 122-SMF/PA/PC 122 | 10 | 0 | 0 | 0 | 0 |
| 123-SMF/PA/PC 123 | 2 | 0 | 0 | 0 | 0 |
| 124-SMF/PA/PC 124 | 10 | 0 | 0 | 0 | 0 |
| 126-SMF/PA/PC 126 | 2 | 6 | 0 | 0 | 0 |
| 127-SMF/PA/PC 127 | 10 | 0 | 0 | 0 | 0 |
| 128-SMF/PA/PC 128 | 4 | 0 | 0 | 0 | 0 |
| 129-SMF/PA/PC 129 | 9 | 0 | 0 | 0 | 0 |
| 130-SMF/PA/PC 130 | 9 | 0 | 0 | 0 | 0 |
| 131-SMF/PA/PC 131 | 6 | 0 | 0 | 0 | 0 |
| 132-SMF/PA/PC 132 | 11 | 0 | 0 | 0 | 0 |
| 133-SMF/PA/PC 133 | 8 | 0 | 0 | 0 | 0 |
| 134-SMF/PA/PC 134 | 3 | 0 | 0 | 0 | 0 |
| 135-SMF/PA/PC 135 | 13 | 0 | 0 | 0 | 0 |
| 136-SMF/PA/PC 136 | 25 | 0 | 0 | 0 | 0 |
| 137-SMF/PA/PC 137 | 4 | 7 | 0 | 0 | 0 |
| 138-SMF/PA/PC 138 | 5 | 0 | 0 | 0 | 0 |
| 139-SMF/PA/PC 139 | 22 | 0 | 0 | 0 | 0 |
| 140-SMF/PA/PC 140 | 13 | 0 | 0 | 0 | 0 |
| 141-SMF/PA/PC 141 | 23 | 0 | 0 | 0 | 0 |
| 142-SMF/PA/PC 142 | 21 | 0 | 0 | 0 | 0 |
| 143-SMF/PA/PC 143 | 24 | 0 | 0 | 0 | 0 |
| 144-SMF/PA/PC 144 | 28 | 0 | 0 | 0 | 0 |
| 145-SMF/PA/PC 145 | 7 | 0 | 0 | 0 | 0 |
| 146-SMF/PA/PC 146 | 10 | 0 | 0 | 0 | 0 |
| 147-SMF/PA/PC 147 | 6 | 0 | 0 | 0 | 0 |
| 148-SMF/PA/PC 148 | 8 | 0 | 0 | 0 | 0 |
| 149-SMF/PA/PC 149 | 9 | 0 | 0 | 0 | 0 |
| 150-SMF/PA/PC 150 | 8 | 0 | 0 | 0 | 0 |
| 151-SMF/PA/PC 151 | 10 | 0 | 0 | 0 | 0 |
| 152-SMF/PA/PC 152 | 8 | 0 | 0 | 0 | 0 |
| 153-SMF/PA/PC 153 | 8 | 0 | 0 | 0 | 0 |
| 154-SMF/PA/PC 154 | 9 | 0 | 0 | 0 | 0 |
| 155-SMF/PA/PC 155 | 8 | 0 | 0 | 0 | 0 |
| 156-SMF/PA/PC 156 | 5 | 0 | 0 | 0 | 0 |
| 157-SMF/PA/PC 157 | 7 | 0 | 0 | 0 | 0 |
| 158-SMF/PA/PC 158 | 7 | 0 | 0 | 0 | 0 |
| 159-SMF/PA/PC 159 | 9 | 0 | 0 | 0 | 0 |
| 160-SMF/PA/PC 160 | 10 | 0 | 0 | 0 | 0 |
| 161-SMF/PA/PC 161 | 0 | 0 | 0 | 0 | 0 |
| 162-SMF/PA/PC 162 | 2 | 0 | 0 | 0 | 0 |
| 163-SMF/PA/PC 163 | 8 | 0 | 0 | 0 | 0 |
| 164-SMF/PA/PC 164 | 5 | 0 | 0 | 0 | 0 |
| 165-SMF/PA/PC 165 | 7 | 0 | 0 | 0 | 0 |
| 166-SMF/PA/PC 166 | 0 | 0 | 0 | 0 | 0 |
| 167-SMF/PA/PC 167 | 0 | 0 | 0 | 0 | 0 |
| 168-SMF/PA/PC 168 | 10 | 0 | 0 | 0 | 0 |
| 170-SMF/PA/PC 170 | 8 | 0 | 0 | 0 | 0 |
| 171-SMF/PA/PC 171 | 1 | 0 | 0 | 0 | 0 |
| 172-SMF/PA/PC 172 | 10 | 0 | 0 | 0 | 0 |
| 173-SMF/PA/PC 173 | 9 | 0 | 0 | 0 | 0 |
| 174-SMF/PA/PC 174 | 0 | 0 | 0 | 0 | 0 |
| 175-SMF/PA/PC 175 | 5 | 0 | 0 | 0 | 0 |
| 176-SMF/PA/PC 176 | 9 | 0 | 0 | 0 | 0 |
| 177-SMF/PA/PC 177 | 6 | 0 | 0 | 0 | 0 |
| 178-SMF/PA/PC 178 | 0 | 0 | 0 | 0 | 0 |
| 179-SMF/PA/PC 179 | 0 | 0 | 0 | 0 | 0 |
| 180-SMF/PA/PC 180 | 1 | 0 | 0 | 0 | 0 |
| 181-SMF/PA/PC 181 | 10 | 0 | 0 | 0 | 0 |
| 182-SMF/PA/PC 182 | 10 | 0 | 0 | 0 | 0 |
| 183-SMF/PA/PC 183 | 4 | 0 | 0 | 0 | 1 |
| 184-SMF/PA/PC 184 | 12 | 0 | 0 | 0 | 0 |
| 186-SMF/PA/PC 186 | 0 | 0 | 0 | 0 | 0 |
| 187-SMF/PA/PC 187 | 6 | 0 | 0 | 0 | 0 |
| 188-SMF/PA/PC 188 | 6 | 0 | 0 | 0 | 0 |
| 189-SMF/PA/PC 189 | 2 | 5 | 0 | 0 | 0 |
| 190-SMF/PA/PC 190 | 6 | 0 | 0 | 0 | 0 |
| 191-SMF/PA/PC 191 | 0 | 0 | 0 | 0 | 0 |
| 192-SMF/PA/PC 192 | 8 | 0 | 0 | 0 | 0 |
| 193-SMF/PA/PC 193 | 1 | 0 | 0 | 0 | 0 |
| 194-SMF/PA/PC 194 | 8 | 0 | 0 | 0 | 0 |
| 195-SMF/PA/PC 195 | 9 | 0 | 0 | 0 | 0 |
| 197-SMF/PA/PC 197 | 14 | 0 | 0 | 0 | 0 |
| 198-SMF/PA/PC 198 | 3 | 0 | 0 | 0 | 0 |
| 199-SMF/PA/PC 199 | 4 | 0 | 0 | 0 | 0 |
| 200-SMF/PA/PC 200 | 0 | 0 | 0 | 0 | 0 |
| 202-SMF/PA/PC 202 | 0 | 0 | 0 | 0 | 0 |
| 203-SMF/PA/PC 203 | 8 | 0 | 0 | 0 | 0 |
| 204-SMF/PA/PC 204 | 7 | 0 | 0 | 0 | 0 |
| 205-SMF/PA/PC 205 | 5 | 0 | 0 | 0 | 0 |
| 207-SMF/PA/PC 207 | 0 | 0 | 0 | 0 | 0 |
| 208-SMF/PA/PC 208 | 5 | 0 | 0 | 0 | 0 |
| 209-SMF/PA/PC 209 | 0 | 0 | 0 | 0 | 0 |
| 210-SMF/PA/PC 210 | 6 | 0 | 0 | 0 | 0 |
| 212-SMF/PA/PC 212 | 4 | 0 | 0 | 0 | 0 |
| 213-SMF/PA/PC 213 | 0 | 0 | 0 | 0 | 0 |
| 215-SMF/PA/PC 215 | 0 | 0 | 0 | 0 | 0 |
| 216-SMF/PA/PC 216 | 0 | 0 | 0 | 0 | 0 |
| 217-SMF/PA/PC 217 | 0 | 0 | 0 | 0 | 0 |
| 219-SMF/PA/PC 219 | 11 | 0 | 0 | 0 | 0 |
| 220-SMF/PA/PC 220 | 1 | 0 | 0 | 0 | 0 |
| 221-SMF/PA/PC 221 | 0 | 4 | 0 | 0 | 0 |
| 223-SMF/PA/PC 223 | 10 | 0 | 0 | 0 | 0 |
| 224-SMF/PA/PC 224 | 6 | 0 | 0 | 0 | 0 |
| 225-SMF/PA/PC 225 | 0 | 0 | 0 | 0 | 0 |
| 226-SMF/PA/PC 226 | 0 | 0 | 0 | 0 | 0 |
| 227-SMF/PA/PC 227 | 0 | 0 | 0 | 0 | 0 |
| 228-SMF/PA/PC 228 | 0 | 0 | 0 | 0 | 0 |
| 229-SMF/PA/PC 229 | 0 | 0 | 0 | 0 | 0 |
| 230-SMF/PA/PC 230 | 0 | 0 | 0 | 0 | 0 |
| 232-SMF/PA/PC 232 | 10 | 0 | 0 | 0 | 0 |
| 234-SMF/PA/PC 234 | 28 | 0 | 0 | 0 | 0 |
| 235-SMF/PA/PC 235 | 32 | 0 | 0 | 0 | 0 |
| 236-SMF/PA/PC 236 | 31 | 0 | 0 | 0 | 0 |
| 237-SMF/PA/PC 237 | 31 | 0 | 0 | 0 | 0 |
| 238-SMF/PA/PC 238 | 16 | 0 | 0 | 0 | 0 |
| 239-SMF/PA/PC 239 | 27 | 0 | 0 | 0 | 0 |
| 240-SMF/PA/PC 240 | 24 | 0 | 0 | 0 | 0 |
| 241-SMF/PA/PC 241 | 29 | 0 | 0 | 0 | 0 |
| 242-SMF/PA/PC 242 | 27 | 0 | 0 | 0 | 0 |
| 243-SMF/PA/PC 243 | 26 | 0 | 0 | 0 | 0 |
| 244-SMF/PA/PC 244 | 16 | 0 | 0 | 0 | 0 |
| 245-SMF/PA/PC 245 | 4 | 20 | 0 | 0 | 0 |
| 246-SMF/PA/PC 246 | 21 | 0 | 1 | 0 | 0 |
| 247-SMF/PA/PC 247 | 16 | 0 | 0 | 0 | 0 |
| 249-SMF/PA/PC 249 | 20 | 0 | 0 | 0 | 0 |
| 250-SMF/PA/PC 250 | 18 | 0 | 0 | 0 | 0 |
| 251-SMF/PA/PC 251 | 12 | 0 | 0 | 0 | 0 |
| 252-SMF/PA/PC 252 | 21 | 0 | 0 | 0 | 0 |
| 253-SMF/PA/PC 253 | 5 | 0 | 0 | 0 | 0 |
| 254-SMF/PA/PC 254 | 20 | 0 | 0 | 0 | 0 |
| 256-SMF/PA/PC 256 | 0 | 13 | 0 | 0 | 0 |
| 257-SMF/PA/PC 257 | 8 | 0 | 2 | 0 | 2 |
| 258-SMF/PA/PC 258 | 10 | 0 | 0 | 0 | 5 |
| 260-SMF/PA/PC 260 | 10 | 0 | 0 | 0 | 0 |
| 261-SMF/PA/PC 261 | 9 | 0 | 0 | 0 | 0 |
| 262-SMF/PA/PC 262 | 9 | 0 | 0 | 0 | 0 |
| 263-SMF/PA/PC 263 | 10 | 0 | 0 | 0 | 0 |
| 264-SMF/PA/PC 264 | 0 | 0 | 0 | 0 | 0 |
| 265-SMF/PA/PC 265 | 0 | 0 | 0 | 0 | 0 |
| 266-SMF/PA/PC 266 | 3 | 0 | 0 | 0 | 0 |
| 267-SMF/PA/PC 267 | 10 | 0 | 0 | 0 | 0 |
| 268-SMF/PA/PC 268 | 10 | 0 | 0 | 0 | 0 |
| 269-SMF/PA/PC 269 | 10 | 0 | 1 | 0 | 0 |
| 270-SMF/PA/PC 270 | 4 | 0 | 0 | 0 | 0 |
| 271-SMF/PA/PC 271 | 10 | 0 | 0 | 0 | 0 |
| 272-SMF/PA/PC 272 | 8 | 0 | 0 | 0 | 0 |
| 274-SMF/PA/PC 274 | 7 | 0 | 0 | 0 | 0 |
| 275-SMF/PA/PC 275 | 0 | 0 | 0 | 0 | 0 |
| 276-SMF/PA/PC 276 | 14 | 0 | 0 | 0 | 0 |
| 277-SMF/PA/PC 277 | 10 | 0 | 0 | 0 | 0 |
| 278-SMF/PA/PC 278 | 11 | 0 | 0 | 0 | 0 |
| 279-SMF/PA/PC 279 | 0 | 0 | 0 | 0 | 0 |
| 280-SMF/PA/PC 280 | 0 | 0 | 0 | 0 | 0 |
| 282-SMF/PA/PC 282 | 0 | 0 | 0 | 0 | 0 |
| 283-SMF/PA/PC 283 | 10 | 0 | 0 | 0 | 0 |
| 284-SMF/PA/PC 284 | 4 | 0 | 0 | 0 | 0 |
| 285-SMF/PA/PC 285 | 10 | 0 | 0 | 0 | 0 |
| 286-SMF/PA/PC 286 | 10 | 0 | 0 | 0 | 0 |
| 287-SMF/PA/PC 287 | 6 | 0 | 0 | 0 | 0 |
| 288-SMF/PA/PC 288 | 9 | 0 | 0 | 0 | 0 |
| 289-SMF/PA/PC 289 | 0 | 0 | 0 | 0 | 0 |
| 290-SMF/PA/PC 290 | 6 | 0 | 0 | 2 | 4 |
| 291-SMF/PA/PC 291 | 8 | 0 | 0 | 0 | 0 |
| 292-SMF/PA/PC 292 | 2 | 5 | 0 | 0 | 0 |
| 293-SMF/PA/PC 293 | 10 | 0 | 0 | 0 | 0 |
| 294-SMF/PA/PC 294 | 2 | 0 | 0 | 0 | 0 |
| 295-SMF/PA/PC 295 | 10 | 0 | 0 | 0 | 0 |
| 296-SMF/PA/PC 296 | 8 | 2 | 0 | 0 | 0 |
| 297-SMF/PA/PC 297 | 1 | 0 | 0 | 0 | 0 |
| 298-SMF/PA/PC 298 | 5 | 0 | 0 | 0 | 0 |
| 299-SMF/PA/PC 299 | 0 | 0 | 0 | 0 | 0 |
| 300-SMF/PA/PC 300 | 10 | 0 | 0 | 1 | 0 |
| 301-SMF/PA/PC 301 | 2 | 0 | 0 | 0 | 0 |
| 302-SMF/PA/PC 302 | 0 | 0 | 0 | 0 | 0 |
| 303-SMF/PA/PC 303 | 0 | 0 | 0 | 0 | 0 |
| 304-SMF/PA/PC 304 | 0 | 0 | 0 | 0 | 0 |
| 305-SMF/PA/PC 305 | 0 | 0 | 0 | 0 | 0 |
| 306-SMF/PA/PC 306 | 10 | 0 | 0 | 0 | 0 |
| 307-SMF/PA/PC 307 | 8 | 0 | 0 | 0 | 0 |
| 308-SMF/PA/PC 308 | 8 | 0 | 0 | 1 | 0 |
| 309-SMF/PA/PC 309 | 0 | 0 | 0 | 0 | 0 |
| 310-SMF/PA/PC 310 | 4 | 0 | 0 | 0 | 0 |
| 312-SMF/PA/PC 312 | 7 | 0 | 0 | 0 | 0 |
| 314-SMF/PA/PC 314 | 10 | 0 | 0 | 0 | 0 |
| 315-SMF/PA/PC 315 | 6 | 0 | 0 | 0 | 0 |
| 317-SMF/PA/PC 317 | 5 | 0 | 0 | 0 | 0 |
| 318-SMF/PA/PC 318 | 0 | 0 | 0 | 0 | 0 |
| 319-SMF/PA/PC 319 | 9 | 0 | 0 | 0 | 0 |
| 320-SMF/PA/PC 320 | 0 | 0 | 0 | 0 | 0 |
| 321-SMF/PA/PC 321 | 10 | 0 | 0 | 0 | 0 |
| 322-SMF/PA/PC 322 | 6 | 0 | 0 | 0 | 0 |
| 323-SMF/PA/PC 323 | 8 | 0 | 0 | 0 | 0 |
| 324-SMF/PA/PC 324 | 9 | 0 | 0 | 0 | 0 |
| 325-SMF/PA/PC 325 | 9 | 0 | 0 | 0 | 4 |
| 326-SMF/PA/PC 326 | 0 | 0 | 0 | 0 | 0 |
| 327-SMF/PA/PC 327 | 8 | 0 | 0 | 0 | 0 |
| 328-SMF/PA/PC 328 | 0 | 0 | 0 | 0 | 0 |
| 329-SMF/PA/PC 329 | 7 | 0 | 0 | 0 | 0 |
| 330-SMF/PA/PC 330 | 3 | 0 | 0 | 0 | 0 |
| 331-SMF/PA/PC 331 | 3 | 0 | 0 | 0 | 0 |
| 332-SMF/PA/PC 332 | 10 | 0 | 0 | 0 | 0 |
| 333-SMF/PA/PC 333 | 8 | 0 | 0 | 0 | 0 |
| 334-SMF/PA/PC 334 | 10 | 0 | 0 | 0 | 0 |
| 335-SMF/PA/PC 335 | 0 | 0 | 0 | 0 | 0 |
| 336-SMF/PA/PC 336 | 10 | 0 | 0 | 0 | 0 |
| 337-SMF/PA/PC 337 | 8 | 0 | 0 | 0 | 0 |
| 338-SMF/PA/PC 338 | 6 | 0 | 0 | 0 | 0 |
| 339-SMF/PA/PC 339 | 2 | 4 | 0 | 0 | 0 |
| 340-SMF/PA/PC 340 | 1 | 0 | 0 | 0 | 0 |
| 341-SMF/PA/PC 341 | 0 | 8 | 0 | 0 | 0 |
| 342-SMF/PA/PC 342 | 4 | 18 | 0 | 0 | 0 |
| 343-SMF/PA/PC 343 | 30 | 0 | 0 | 0 | 6 |
| 344-SMF/PA/PC 344 | 4 | 20 | 0 | 0 | 0 |
| 345-SMF/PA/PC 345 | 4 | 20 | 0 | 0 | 0 |
| 346-SMF/PA/PC 346 | 24 | 0 | 0 | 0 | 0 |
| 347-SMF/PA/PC 347 | 29 | 0 | 0 | 0 | 0 |
| 348-SMF/PA/PC 348 | 0 | 0 | 0 | 0 | 0 |
| 349-SMF/PA/PC 349 | 30 | 0 | 0 | 0 | 0 |
| 350-SMF/PA/PC 350 | 14 | 0 | 0 | 0 | 0 |
| 351-SMF/PA/PC 351 | 0 | 19 | 0 | 0 | 0 |
| 352-SMF/PA/PC 352 | 25 | 0 | 0 | 0 | 0 |
| 353-SMF/PA/PC 353 | 30 | 0 | 0 | 1 | 3 |
| 354-SMF/PA/PC 354 | 4 | 20 | 0 | 0 | 0 |
| 355-SMF/PA/PC 355 | 7 | 16 | 0 | 0 | 0 |
| 356-SMF/PA/PC 356 | 1 | 19 | 0 | 0 | 0 |
| 357-SMF/PA/PC 357 | 8 | 14 | 0 | 0 | 0 |
| 358-SMF/PA/PC 358 | 25 | 0 | 0 | 0 | 0 |
| 359-SMF/PA/PC 359 | 24 | 0 | 0 | 0 | 0 |
| 360-SMF/PA/PC 360 | 29 | 0 | 0 | 0 | 0 |
| 361-SMF/PA/PC 361 | 19 | 0 | 0 | 0 | 0 |
| 362-SMF/PA/PC 362 | 6 | 0 | 0 | 0 | 0 |
| 363-SMF/PA/PC 363 | 5 | 0 | 0 | 0 | 0 |
| 364-SMF/PA/PC 364 | 9 | 0 | 0 | 0 | 0 |
| 365-SMF/PA/PC 365 | 6 | 0 | 0 | 0 | 0 |
| 366-SMF/PA/PC 366 | 10 | 0 | 0 | 0 | 0 |
| 367-SMF/PA/PC 367 | 11 | 0 | 0 | 0 | 0 |
| 368-SMF/PA/PC 368 | 8 | 0 | 0 | 0 | 0 |
| 369-SMF/PA/PC 369 | 13 | 0 | 0 | 0 | 0 |
| 370-SMF/PA/PC 370 | 20 | 0 | 0 | 0 | 0 |
| 371-SMF/PA/PC 371 | 4 | 0 | 0 | 0 | 0 |
| 372-SMF/PA/PC 372 | 32 | 0 | 0 | 0 | 0 |
| 373-SMF/PA/PC 373 | 30 | 0 | 0 | 0 | 0 |
| 374-SMF/PA/PC 374 | 26 | 0 | 0 | 0 | 0 |
| 375-SMF/PA/PC 375 | 4 | 18 | 0 | 0 | 0 |
| 376-SMF/PA/PC 376 | 0 | 20 | 0 | 0 | 0 |
| 378-SMF/PA/PC 378 | 29 | 0 | 0 | 0 | 0 |
| 379-SMF/PA/PC 379 | 9 | 0 | 0 | 0 | 0 |
| 380-SMF/PA/PC 380 | 7 | 0 | 0 | 0 | 0 |
| 381-SMF/PA/PC 381 | 23 | 0 | 0 | 0 | 0 |
| 382-SMF/PA/PC 382 | 23 | 0 | 0 | 0 | 0 |
| 384-SMF/PA/PC 384 | 18 | 0 | 0 | 0 | 0 |
| 385-SMF/PA/PC 385 | 31 | 0 | 0 | 0 | 0 |
| 386-SMF/PA/PC 386 | 7 | 0 | 0 | 0 | 0 |
| 386-SMF/PA/PC 386-A | 6 | 0 | 0 | 0 | 0 |
| 387-SMF/PA/PC 387 | 9 | 0 | 0 | 0 | 1 |
| 388-SMF/PA/PC 388 | 8 | 0 | 0 | 0 | 0 |
| 389-SMF/PA/PC 389 | 3 | 0 | 0 | 0 | 0 |
| 390-SMF/PA/PC 390 | 4 | 0 | 0 | 0 | 0 |
| 391-SMF/PA/PC 391 | 1 | 0 | 0 | 0 | 0 |
| 392-SMF/PA/PC 392 | 10 | 0 | 0 | 0 | 0 |
| 394-SMF/PA/PC 394 | 32 | 0 | 0 | 0 | 1 |
| 395-SMF/PA/PC 395 | 8 | 0 | 0 | 0 | 2 |
| 396-SMF/PA/PC 396 | 29 | 0 | 0 | 0 | 0 |
| 397-SMF/PA/PC 397 | 19 | 0 | 0 | 0 | 0 |
| 398-SMF/PA/PC 398 | 3 | 13 | 0 | 0 | 0 |
| 399-SMF/PA/PC 399 | 7 | 0 | 0 | 0 | 0 |
| 400-SMF/PA/PC 400 | 4 | 0 | 0 | 0 | 0 |
| 402-SMF/PA/PC 402 | 32 | 0 | 0 | 0 | 0 |
| 404-SMF/PA/PC 404 | 32 | 0 | 0 | 1 | 0 |
| 406-SMF/PA/PC 406 | 32 | 0 | 0 | 0 | 0 |
