# Supplementary material for: Earliest evidence of caries lesion in hominids reveal sugar-rich diet for a Middle Miocene dryopithecine from Europe
Source: PLoS One. 2018 Aug 30;13(8):e0203307. doi: 10.1371/journal.pone.0203307 (PMC6117023; doi:10.1371/journal.pone.0203307)
Supplement: S2 Table — (DOCX) [file pone.0203307.s002.docx]

**S2 Table.** Potential melliferous plants of the Lavanttal flora [1-5].

| Genus | Number of species | Flowering month [6] | References |
| --- | --- | --- | --- |
| *Abies* | 3 | 4,5 | [7-9] |
| *Acer* | 4 | 4,5,6 | [7] |
| *Alnus* | 2 | 3,4 | [7] |
| *Arbutus* | 1 | 10,11, 12, 1,2,3 | [7] |
| *Betula* | 2 | 4,5 | [7] |
| *Carya* | 1 | 5,6 | [10] |
| *Castanea* | 1 | 6,7 | [7] |
| *Corylus* | 1 | 2,3 | [7] |
| *Elaeagnus* | 1 | 5,6 | [7] |
| *Fagus* | 1 | 4,5 | [7, 8] |
| *Hamamelis* | 1 | 12,1,2,3 | [11, 12] |
| *Hedera* | 1 | 9,10 | [7] |
| *Juglans* | 1 | 4,5 | [7] |
| *Larix* | 1 | 3,4,5 | [9] |
| *Ostrya* | 1 | 4,5,6 | [7] |
| *Parthenocissus* | 4 | 6,7 | [7] |
| *Picea* | 1 | 5,6 | [8, 9] |
| *Pinus* | 4 | 5,6 | [7, 9] |
| *Prunus* | 2 | 3,4 | [7, 8] |
| *Quercus* | 7 | 4,5 | [7, 8] |
| *Salix* | 2 | 3,4 | [7, 8, 10] |
| *Symplocos* | 1 | 5,6 | [13] |
| *Tilia* | 1 | 6,7 | [7, 8] |
| *Ulmus* | 2 | 3,4 | [7] |
| Total potential  melliferous species | 46 |  |  |

**References**

1. Klaus W. Zur Mikroflora des Unter-Sarmat am Alpen-Südostrand. Beiträge zur Paläontologie von Österreich. 1984;11:289 - 419.

2. Grímsson F, Zetter R, Baal C. Combined LM and SEM study of the Middle Miocene (Sarmatian) palynoflora from the Lavanttal Basin, Austria: Part I. Bryophyta, Lycopodiophyta, Pteridophyta, Ginkgophyta, and Gnetophyta. Grana. 2011;50(2):102-28.

3. Grímsson F, Zetter R. Combined LM and SEM study of the Middle Miocene (Sarmatian) palynoflora from the Lavanttal Basin, Austria: Part II. Pinophyta (Cupressaceae, Pinaceae and Sciadopityaceae). Grana. 2011;50(4):262-310.

4. Grímsson F, Meller B, Bouchal JM, Zetter R. Combined LM and SEM study of the middle Miocene (Sarmatian) palynoflora from the Lavanttal Basin, Austria: part III. Magnoliophyta 1 – Magnoliales to Fabales. Grana. 2015;54(2):85-128.

5. Grímsson F, Grimm GW, Meller B, Bouchal JM, Zetter R. Combined LM and SEM study of the middle Miocene (Sarmatian) palynoflora from the Lavanttal Basin, Austria: part IV. Magnoliophyta 2 – Fagales to Rosales. Grana. 2015;55(2):101-63.

6. Gurk C, Hepp C. <http://www.baumkunde.de/> [accessed 20.6.2017].

7. Tashev AN, Velinova ES, Tsavkov EI. Melliferous plants of Bulgarian dendroflora. Phytologia Balcanica. 2015;21(3):295 - 302.

8. Antonie I. The biodiversity of the melliferous plants in the surroundings of the town Sebes (Alba County) and their economical importance. Scientific Papers Series Management, Economic Engineering in Agriculture and Rural Development. 2014;14(4).

9. Carter C, Maslen NR. Conifer Lachnids. London: Her Majesty`s Stationery Office.

10. Dongock DN, Tchoumboue J, Ricciardelli D'Albore G, Youmbi E, Pinta YJ. Spectrum of melliferous plants used byApis mellifera adansoniiin the Sudano‐Guinean western highlands of Cameroon. Grana. 2007;46(2):123-8.

11. Anderson GJ, Hill JD. Many to flower, few to fruit: the reproductive biology of Hamamelis virginiana (Hamamelidaceae). American journal of botany. 2002;89(1):67-78.

12. Schedl W. Blütenökologische Beobachtungen an der Chinesischen Zaubernuss (Hamamelis mollis OLIV.) Hamamlidaceae) im Botanischen Garten in Innsbruck (Österreich). Ber nat-med Verein Inssbruck. 1992;79:145 - 52.

13. Kumar N, Jangwan JS. Phytoconstituents of symplocos paniculata (leaves). Journal of Current Chemical & Pharmaceutical Sciences. 2012;2(1):76 - 80.
